# Charging the Future with Pioneering MXenes: Scalable 2D Materials for Next-Generation Batteries

**DOI:** 10.3390/nano15141089

**Published:** 2025-07-14

**Authors:** William Coley, Amir-Ali Akhavi, Pedro Pena, Ruoxu Shang, Yi Ma, Kevin Moseni, Mihrimah Ozkan, Cengiz S. Ozkan

**Affiliations:** 1 Materials Science and Engineering Program, University of California Riverside, Riverside, CA 92521, USA; wcole001@ucr.edu (W.C.); aakha001@ucr.edu (A.-A.A.); ruoxu.shang@email.ucr.edu (R.S.); kmose003@ucr.edu (K.M.); 2 Department of Chemistry, University of California Riverside, Riverside, CA 92521, USA; pedro.a.penarodriguez@email.ucr.edu; 3 Department of Mechanical Engineering, University of California Riverside, Riverside, CA 92521, USA; yma102@ucr.edu; 4 Department of Electrical and Computer Engineering, University of California Riverside, Riverside, CA 92521, USA

**Keywords:** MXene, precursor materials, rechargeable battery, sustainability, scalability

## Abstract

MXenes, a family of two-dimensional carbide and nitride nanomaterials, have demonstrated significant promise across various technological domains, particularly in energy storage applications. This review critically examines scalable synthesis techniques for MXenes and their potential integration into next-generation rechargeable battery systems. We highlight both top-down and emerging bottom-up approaches, exploring their respective efficiencies, environmental impacts, and industrial feasibility. The paper further discusses the electrochemical behavior of MXenes in lithium-ion, sodium-ion, and aluminum-ion batteries, as well as their multifunctional roles in solid-state batteries—including as electrodes, additives, and solid electrolytes. Special emphasis is placed on surface functionalization, interlayer engineering, and ion transport properties. We also compare MXenes with conventional graphite anodes, analyzing their gravimetric and volumetric performance potential. Finally, challenges such as diffusion kinetics, power density limitations, and scalability are addressed, providing a comprehensive outlook on the future of MXenes in sustainable energy storage technologies.

## 1. Introduction

Modern renewable energy technologies—including solar, wind, and geothermal—have significantly expanded global energy generation capacity. However, the lack of adequate large-scale energy storage solutions remains a critical bottleneck in fully realizing the potential of renewable sources. While commercial battery systems such as the Tesla Powerwall [1] have enabled localized energy storage for residential and commercial users, these systems are insufficient to address grid-scale demands. For instance, California frequently compensates neighboring states to offload surplus energy due to overgeneration from wind and solar [2]. This highlights a mismatch between peak generation hours and consumption patterns. During late afternoon and early evening peak demand periods, stored energy is often inadequate, necessitating the use of carbon-emitting power plants to balance the grid.

As nations pursue carbon neutrality goals, a substantial increase in energy storage capacity is essential to support a higher share of renewables in the energy mix [3]. This necessitates upgrades to existing grid infrastructure to accommodate fluctuations in energy production and consumption. Energy generated during peak sunlight or wind periods must be captured and stored for redistribution during demand surges. The International Renewable Energy Agency (IRENA) projected in 2017 that global non-hydroelectric energy storage capacity would increase from approximately 162 GWh in 2017 to between 5821 GWh and 8426 GWh by 2030 [3]. Achieving such growth requires innovative approaches, not only in electrochemical systems but also in complementary modalities such as thermal and mechanical energy storage.

The emergence of MXenes has introduced a transformative platform for addressing these storage challenges. Following the pioneering work of Naguib et al. in 2011, it was demonstrated that layered transition metal carbides and nitrides of the MAX phase (general formula M_n+1_AX_n_) could be selectively etched to remove the A layer, yielding stable two-dimensional materials now known as MXenes [4]. The conversion of Ti_3_AlC_2_ to Ti_3_C_2_Tx marked a significant milestone in materials science, leading to the rapid development of this novel class of 2D nanomaterials [4,5,6].

Since their discovery, a wide variety of MXenes have been synthesized from parent MAX phase precursors. Transition metals used in the M-layer include Y, Ti, V, Cr, Zr, Nb, Mo, Hf, Ta, and W [4]. Theoretical models also predict that metastable MXenes can be formed from Sc, Mn, and most lanthanide series elements [6,7]. While carbon (C) and nitrogen (N) have been experimentally validated as the X component, ab initio calculations suggest boron (B) may also be viable [6]. With over 20 documented compositions and many more hypothetical ones (e.g., Y_2_C, V_2_N), the MXene family offers expansive opportunities for exploration and application [4]. This versatility is further enhanced by variations in structure, such as the number of M layers per unit cell.

Given this structural and compositional diversity, MXenes are poised for applications across a broad spectrum of technologies [8,9,10]. This review specifically examines their role in energy storage, particularly in rechargeable batteries. Emphasis is placed on cost-effective, scalable synthesis routes and the incorporation of sustainable, environmentally benign precursor materials. MXenes have shown potential as both anode and cathode materials in lithium-, sodium-, and potassium-ion battery systems [5,11,12,13,14]. Their tunable surface chemistries, high electrical conductivities, and capacity for ion intercalation make them attractive candidates for next-generation batteries.

The careful selection of M and X elements significantly impacts the resulting MXene’s electrochemical properties. Research has predominantly focused on lightweight transition metals such as Ti, V, and Cr [7], which provide favorable gravimetric capacities and structural stability. Concurrently, there is growing interest in MXenes synthesized from more environmentally abundant and less toxic elements such as Mo and W. These efforts reflect a broader shift towards the development of high-performance, sustainable energy storage materials that align with global clean energy goals.

## 2. MXenes as a Prospective Replacement for Graphite in Energy Storage Systems

Graphite has served as the predominant commercial anode material in lithium-ion (Li^+^) batteries since the 1990s. Its widespread adoption, coupled with decades of technological refinement, has brought graphite to a point of industrial and performance maturity. Consequently, incremental improvements in its performance have plateaued, prompting the exploration of alternative materials such as silicon (Si) and two-dimensional (2D) transition metal carbides and nitrides—collectively known as MXenes. The established industrial infrastructure for graphite production has resulted in relatively low market prices due to standardized and optimized processing techniques. However, graphite is not only essential for battery technologies; it is also a critical material in the metallurgical sector, particularly for steel production. As such, its market price is influenced by a complex interplay of factors, including supply–demand dynamics, production constraints, and geopolitical events.

For instance, in the latter half of 2017, graphite prices surged by 30–40%, driven by heightened demand from the steel and battery industries, exacerbated by environmental and regulatory challenges affecting Chinese production [15]. By early 2018, the price of uncoated spherical graphite increased by an average of USD 375 per ton amid continued supply instability from China [16]. Historically, graphite prices have demonstrated substantial volatility. In 2017, large flake graphite reached USD 1200 per ton, still significantly lower than the 2012 peak of USD 2800 per ton [15]. Similarly, in 2013, prices for 94% purity, +80 mesh flake graphite peaked at USD 1400 per ton—30% higher than pre-recession levels but down from the 2011 high of USD 2500 per ton. In Sri Lanka, vein graphite prices have remained stable since 2012, with 99.1% carbon, +1 mesh graphite priced between USD 2750 and USD 2850 per ton, while amorphous graphite has exhibited a declining trend over the same period [17].

China has historically dominated the global graphite supply chain, with its exports rising markedly between 2003 and 2012, accompanied by a 43.4% increase in the annual average export price [18]. However, events such as the 2011 European debt crisis caused a drop in import volumes, followed by a sharp rise in export prices. Recently, emerging graphite production hubs in East Africa, particularly in Tanzania and Madagascar, have shown potential to diversify the global supply landscape and reduce dependency on Chinese sources [16]. In summary, graphite pricing is shaped by a multifaceted combination of industrial demand, production bottlenecks, and regional geopolitical developments. Ongoing shifts in global supply chains may help stabilize prices in the long term.

Nevertheless, the strategic importance of graphite has led to its classification as a critical mineral by the U.S. government in 2018 [19,20]. In response, countries are actively pursuing the development of domestic reserves and building critical material stockpiles, which is expected to drive mid-term price increases. In this context, the search for alternative anode materials that are less reliant on geopolitically sensitive supply chains is of paramount importance. MXenes have emerged as a strong candidate to replace graphite in battery applications due to their favorable electrochemical properties—particularly high theoretical gravimetric and volumetric capacities [7].

Graphite’s theoretical gravimetric capacity is approximately 372 mAh/g [21]. In contrast, M_2_X-type MXenes offer theoretical capacities ranging from 526 mAh/g to 141 mAh/g, while M_3_X_2_-type MXenes range from 337 mAh/g to 93 mAh/g [7]. These values suggest that MXenes could potentially replace graphite on a mass basis at a ratio of approximately 1:1.24 to 1:0.25, depending on composition. More notably, the volumetric capacities of MXenes exceed that of graphite by a factor of two to four, which is critical for developing compact, high-energy-density storage devices. Additionally, MXene properties can be finely tuned through engineering of surface terminations and interlayer spacing—strategies that can enhance both ion accessibility and storage capacity [22,23,24,25]. This tunability allows for the customization of MXenes to accommodate various intercalating ions, including lithium, sodium, and aluminum [26,27].

Despite these advantages, several challenges must be addressed before MXenes can serve as a widespread replacement for graphite anodes. These include improving ion diffusion behavior, enhancing rate capability and power density, and mitigating issues related to safety and surface reactivity [28,29,30,31,32]. Nonetheless, ongoing advancements in synthesis methods, material engineering, and surface chemistry offer promising pathways toward overcoming these limitations. As such, MXenes hold considerable potential to address both the material scarcity and performance demands of future large-scale energy storage systems.

## 3. Synthesis of MAX and MXene Materials

The commercial viability of two-dimensional (2D) materials for energy storage applications is fundamentally dependent on the scalability, cost-effectiveness, and environmental impact of their synthesis processes. MXenes, a class of 2D transition metal carbides and nitrides, have emerged as promising alternatives to graphite due to their favorable electrochemical properties and potential for scalable production. To accelerate the commercialization of MXene-based technologies, it is critical to develop infrastructure that supports cost-efficient manufacturing while addressing waste management challenges. Sustainable strategies, including recycling and byproduct valorization, are essential to minimizing the environmental footprint of MXene production.

This section provides an overview of established and emerging synthesis techniques for MXenes, evaluating their advantages and limitations with respect to economic feasibility, scalability, and environmental sustainability. Broadly, MXene synthesis methods can be categorized into three main approaches: (1) top-down etching from a parent MAX phase using methods such as acid etching or molten salt processes; (2) bottom-up deposition of thin films on epitaxial substrates via techniques like chemical vapor deposition (CVD) or plasma-enhanced pulsed laser deposition (PEPLD); and (3) template-assisted solution-phase synthesis, including hydrothermal routes and graphite-templating methods. Each approach presents unique trade-offs in terms of processing complexity, yield, and adaptability for large-scale implementation.

## 4. MAX to MXene Conversion: Acid Etching and Molten Salt Etching Techniques

Among the various synthesis techniques available for MXene production, acid etching remains the most widely employed and established method for fabricating a broad range of MXene compositions [4]. This method exploits the difference in bond strength between the M–X and M–A layers in the MAX phase. While the M–X bonds exhibit a hybrid character—metallic, covalent, and ionic—the M–A bonds are purely metallic in nature [4]. This distinction enables the selective removal of the A-group element (typically aluminum) through chemical etching, resulting in the formation of layered transition metal carbides or nitrides known as MXenes.

MAX phases themselves are a large family of ternary carbides and nitrides with the general formula M_n+1_AX_n_, where M is a transition metal, A is a group 13 or 14 element, and X is either carbon and/or nitrogen. These materials possess a unique combination of ceramic and metallic properties, including high mechanical strength, thermal stability, and electrical conductivity. MAX powders are typically synthesized via high-temperature solid-state reactions, including physical vapor deposition (PVD), chemical vapor transport (CVT), and molten salt methods, with reaction temperatures ranging from 700 °C to 1600 °C [33,34,35]. These powders are not only precursors for MXene synthesis but have also been used directly in lithium-ion battery applications, where particle size has been shown to influence Li^+^ ion storage capacity [33]. Their inherent performance stability has spurred growing interest in their commercialization for energy storage technologies [36].

**Figure 1:**
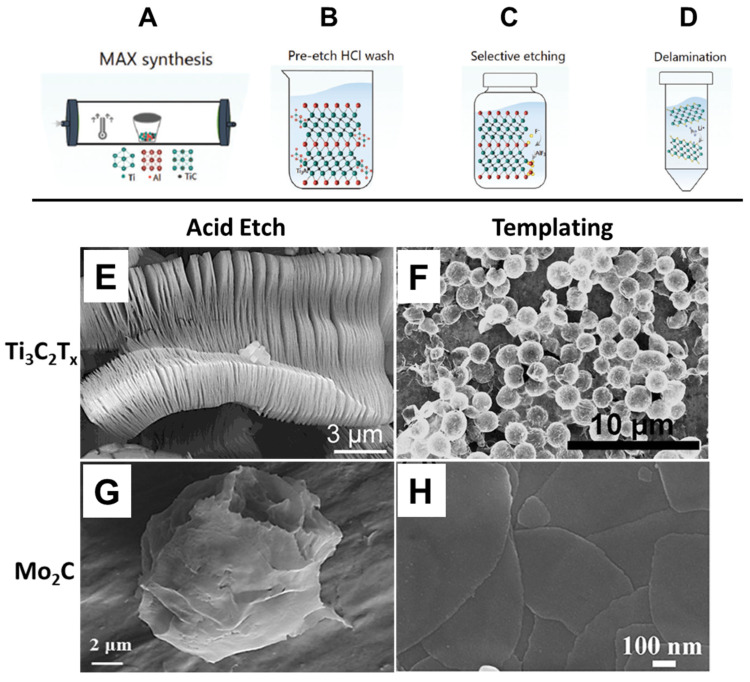
Representative schematic of Ti_3_C_2_T_x_ MXene synthesis and SEM micrographs showing varying morphologies. (**A**–**D**) Representative schematic of the Ti_3_C_2_T_x_ MXene synthesis process [38], as well as SEM micrographs of (**E**,**G**) Ti_3_C_2_T_x_ and (**F**,**H**) Mo_2_C, made via etching of MAX phases and templating. (**A**) Synthesis of Ti_3_AlC_2_ via sintering at 1400 C under inert conditions. (**B**) The recommended pre-etch cleaning step to remove excess TiAl_3_. The powder is first milled below a particle size of 71 um. (**C**) Selective etching of the A layer (Al) in MAX phase to form Ti_3_C_2_T_x_ MXene powders. (**D**) Delamination of the multilayered powder into single Ti_3_C_2_T_x_ flakes. (**E**) Ti_3_C_2_T_x_ layered material [39]. (**F**) Ti_3_C_2_T_x_ nanospheres made by templating using PMMA [40] (**G**) Mo_2_C made by etching Mo_2_Ga_2_C [41] (**H**) Mo_2_C nanosheets made by templating using MoO_2_ nanosheets [42].

The first successful synthesis of MXenes was reported by Naguib et al., who demonstrated selective etching of the aluminum (A-layer) from Ti_3_AlC_2_ using hydrofluoric acid (HF), followed by delamination via sonication [37]. This process produced two-dimensional titanium carbide (Ti_3_C_2_T_x_) with a morphology resembling graphene, giving rise to the term “MXene.” Since then, acid etching has become a foundational top-down method for MXene fabrication. As illustrated in Figure 1A–D, the synthesis typically begins with the preparation of Ti_3_AlC_2_ in a tube furnace, followed by HF-based etching and mechanical delamination to yield Ti_3_C_2_T_x_ nanoflakes. Representative scanning electron microscopy (SEM) images of Ti_3_C_2_T_x_ and Mo_2_C produced via this method are presented in Figure 1E and Figure 1G, respectively, showing the characteristic layered morphology.

To address the safety and environmental concerns associated with hydrofluoric acid, molten salt etching has emerged as a promising alternative. This method mirrors the acid etching approach in principle but replaces HF with oxidative metal chloride or fluoride salts and substitutes the aqueous solvent with a molten salt medium, such as molten chlorides [43] or fluorides [44]. These salts, often used in solid-state form and stored separately, are significantly safer and more stable than liquid HF, reducing handling risks and potential environmental hazards. Molten salt methods thus offer a more sustainable and industrially viable route for producing MXenes, with ongoing research focused on optimizing reaction parameters and expanding the library of accessible compositions.

## 5. Direct Synthesis Methods

In recent years, bottom-up synthesis approaches have gained significant attention as viable alternatives to conventional top-down methods for MXene fabrication. Unlike traditional routes that rely on selective etching of the A layer from MAX phase precursors, direct synthesis techniques involve the direct formation of M–X (metal–carbon or metal–nitrogen) bonds, effectively bypassing the need for ternary precursors and the use of corrosive etchants [45]. This shift enables more sustainable and environmentally friendly production by eliminating A-layer byproducts—typically associated with hazardous waste streams—and streamlining the overall process.

Prominent examples of direct synthesis methods include plasma-enhanced pulsed laser deposition (PEPLD), template-assisted synthesis, and the hydrothermal method. These techniques facilitate the controlled nucleation and growth of MXene phases without the constraints imposed by MAX phase chemistry. Furthermore, direct synthesis routes enable the formation of MXenes incorporating transition metal elements or stoichiometries that are difficult or impossible to achieve via top-down approaches, significantly broadening the range of accessible compositions.

By reducing chemical waste, avoiding hazardous etchants, and enabling tunability in elemental composition, direct synthesis strategies offer a lower carbon footprint and improved scalability, making them particularly attractive for future industrial deployment. Continued advancements in these techniques are expected to play a critical role in realizing greener, more efficient pathways for MXene production.

## 6. Chemical Vapor Deposition

Chemical vapor deposition (CVD) has become a cornerstone technique for the synthesis of two-dimensional (2D) materials, particularly due to its ability to produce highly crystalline and compositionally uniform films. The method operates by reacting volatile gaseous precursors on a heated substrate, enabling the layer-by-layer formation of thin films with precise control over thickness and composition.

Following the discovery of graphene and its remarkable optoelectronic and mechanical properties [46], the search for novel 2D materials expanded to include transition metal dichalcogenides (TMDs), hexagonal boron nitride, and eventually MXenes—layered metal carbides and nitrides. CVD has proven especially valuable in advancing the synthesis of such materials due to its compatibility with various precursors and substrates.

In the context of MXene synthesis, CVD can be broadly categorized into two main strategies:

(1) Conventional CVD, where metal oxides react with reducing gases (e.g., hydrogen or methane) to form epitaxial M–X layers on lattice-matched substrates;
(2) CVD-like processes, in which metal precursors react directly with carbon- or nitrogen-containing gases to yield metal carbides or nitrides.

This technique has played a pivotal role in the fabrication of carbon-based nanostructures, most notably graphene and carbon nanotubes, often synthesized on hexagonal metal foils [47]. Building upon these foundational methods, catalyst-assisted approaches have emerged, exemplified by the work of Xu et al., who successfully synthesized Mo_2_C thin films via CVD [48]. These advances have opened the door to CVD-driven synthesis of diverse MXene compositions, offering new routes that bypass the limitations of traditional etching-based approaches.

Additionally, plasma-enhanced pulsed laser deposition (PEPLD) has been introduced as a complementary method to CVD. While it shares similar deposition mechanics, PEPLD allows for the use of less reactive precursors—such as elemental molybdenum and methane gas—to synthesize MXenes like Mo_2_C [49]. This versatility further expands the toolbox for producing high-quality MXene films with tailored structures and compositions, potentially accelerating their integration into electronic and energy storage devices.

## 7. Templating

Templating synthesis has emerged as a promising bottom-up strategy for producing MXene-like materials, particularly transition metal carbides (TMCs) and transition metal nitrides (TMNs). This approach involves the carbonization or ammoniation of strategically selected precursors deposited onto sacrificial templates. These templates, which often include transition metal oxides and a variety of polymers such as polymethyl methacrylate (PMMA), are engineered to possess crystal structures or lattice parameters that closely resemble the target MXene phases. This structural compatibility facilitates the conversion of oxygen-containing precursors into carbides or nitrides through well-controlled thermal or chemical treatment.

A major advantage of the templating method is its versatility in controlling the morphology of the resulting MXene materials, enabling the synthesis of both nanosheets and more complex architectures such as nanospheres. This adaptability makes it highly attractive for tailoring materials for specific applications, including catalysis, energy storage, and sensing. As illustrated in Figure 1, this method yields distinctive morphologies not typically accessible through top-down approaches such as acid etching. For example, panel F shows Ti_3_C_2_T_x_ nanospheres, while panel H depicts Mo_2_C nanosheets—demonstrating the templating method’s capacity to expand the structural landscape of MXene synthesis.

Template removal and conversion into the final MXene product are typically achieved through thermal techniques, such as annealing or calcination, and vapor-phase methods like chemical vapor deposition (CVD) and hot filament CVD (HFCVD). These processes consume the original template while facilitating the formation of M–X bonds. Although nanosheet morphologies akin to those produced by traditional etching methods are commonly obtained, templating has uniquely enabled the synthesis of spherical and three-dimensional nanostructures, as reported in recent studies [40,50,51]. Such structural diversity significantly broadens the scope of MXene applications and underlines the potential of templating synthesis as a scalable and tunable route for producing next-generation 2D materials.

## 8. Hydrothermal

A promising method for acid-free forming of MXenes is the hydrothermal method. Forging new structures by coupling this one-pot synthesis approach with controlled chemical conditions could yield MXene materials at industry-relevant scales. For example, the reaction follows: metal oxyanions and carbon sources such as simple organic molecules are mixed in water and heated in a Teflon-lined steel autoclave before calcination in a furnace under varying gasses [28]. The precursors are mixed in water, transferred to a hydrothermal vessel, and heated before calcination in a separate furnace. An example of this synthesis method in the literature is demonstrated by Liu et al. [52]. When compared to acid etch synthesis methods, it was possible to significantly reduce the hazardous byproduct production to just the gases released during annealing, significantly lower energy costs by reducing the reaction temperatures, and reduce water usage by removing the post-acid-etch clean-up step. Hydrothermal and acid etching methods show promise in producing battery-quality materials that have the potential to be incorporated into energy storage devices. While similar, they require different reaction conditions, directly affecting the type of waste generated and the overall scalability of the method.

The lower temperatures involved in hydrothermal, CVD (chemical vapor deposition), PEPLD (pulsed laser deposition), and templating methods significantly enhance the sustainability of these processes compared to traditional acid etching. These methods minimize energy consumption by operating at reduced temperatures, thereby reducing the overall carbon footprint associated with material synthesis. Lower-temperature processes are often less harsh, resulting in fewer environmental pollutants and reducing the need for extensive waste management. This contrasts with acid etching, which requires higher energy inputs and generates hazardous byproducts that necessitate careful handling and disposal. Overall, adopting low-temperature techniques represents a crucial step towards more sustainable and environmentally friendly production of MAX powders.

## 9. Analysis of MXene Synthesis Methods

A comprehensive evaluation of MXene synthesis techniques is critical to advancing their use in scalable, sustainable energy storage systems. Key parameters to assess include the stability and quality of the resulting materials, environmental impact (including waste generation and recyclability), and scalability, which encompasses both production yield and process development timelines. While each synthesis method can produce MXenes with properties suitable for a variety of energy storage applications, significant differences exist in efficiency, byproduct generation, and production throughput. As MXenes are considered potential replacements for traditional electrode materials such as graphite, assessing these factors becomes essential to ensure the viability of large-scale deployment.

Table 1 offers a side-by-side comparison of six prominent MXene synthesis approaches, summarizing representative examples, typical reaction conditions, scalability potential, estimated operational costs, and types of waste generated. Notably, the MXene materials produced through these methods are shelf-stable, although they require low-humidity storage environments to prevent oxidation and degradation [53].

Top-down approaches, such as acid etching and molten salt methods, depend on the availability of prefabricated MAX phase precursors. These techniques are well-established and known for producing high-quality MXenes with excellent electrochemical properties [54]. However, they are limited in scalability due to the multi-step synthesis of MAX phases and the use of corrosive chemicals, which necessitate careful waste management. Acid etching, in particular, involves highly acidic reagents that must be neutralized and treated, increasing overall process costs [4]. Incorporating recycling protocols for acidic byproducts could mitigate these issues, but such systems are not yet standard in most labs or pilot facilities.

**Table 1:** Comparison of synthesis methodology requirements.

Synthesis Method	Peak Temperature Required	Additional Furnace Requirements	Furnace Steps Required	Additional Processing Steps	Chemical Etching and Cleaning Steps	Waste Products Produced (Approximate Ranges)
**Hydrothermal Process [55,56]**	800 °C	Hydrogen environment required depending on the material	Low-temperature steps (100–800 °C)	Ball milling steps	Water rinse required	Aqueous waste contaminated with ammonia 0–10 mL per g
**Acid Etch [57,58,59]**	1400–1700 °C	Vacuum or argon environment required	High-temperature steps	Ball milling steps	Chemical etch and cleaning steps required	Aqueous acid waste (HF, HCL, etc.), metal salt waste (depending on acid used, 50–300 mL waste solution per g is common)
**Molten Salt [60,61]**	1400–1700 °C	Vacuum or argon environment required	High-temperature and low-temperature steps	Ball milling steps	Chemical etch and cleaning steps required	Aqueous waste contaminated with metal salt chloride or fluoride waste (depending on the acid used; most papers do not report the exact wash amount)
**CVD [57,62]**	800–1100 °C	Hydrogen environment required depending on the material	Low-temperature steps	Thin film transfer steps	Post-transfer cleanup	Organic solvent waste from the transfer 0–10 mL per g, waste substrate cm^2^ per cm^2^ area of MXene deposit
**PEPLD [49]**	700 °C	Vacuum environment required	Low-temperature steps	Thin film transfer steps	Post transfer cleanup	Organic solvent waste from the transfer, 0–10 mL per g, waste substrate cm^2^ per cm^2^ area of MXene deposit
**Templating Method [63,64]**	900 °C	Hydrogen environment required depending on the material	Low-temperature steps	-	Water rinse required	Aqueous waste contaminated with ammonia and polymer waste 0–10 mL per g, waste substrate cm^2^ per cm^2^ area of MXene deposit

Vapor-phase techniques, such as chemical vapor deposition (CVD) and plasma-enhanced pulsed laser deposition (PEPLD), excel in producing highly crystalline 2D films, making them ideal for precision applications in semiconductors, transparent conductors, on-device capacitors, and photovoltaics. Despite their high material quality, these methods offer relatively low material output per substrate area, which presents a significant limitation for bulk energy storage applications requiring larger material volumes and thicker electrode structures.

Hydrothermal and templating methods, on the other hand, offer simpler processing steps and may provide higher yields, making them attractive for large-scale MXene production. These methods also generate less chemical waste and are more environmentally benign, especially when utilizing commercially available metal precursors or biomass-derived carbon sources. However, limitations include restricted precursor flexibility and the potential for residual templating agents to be incorporated into the final material. Additionally, while these methods may be more sustainable, a detailed life-cycle assessment is needed to fully quantify their environmental benefits.

Across all synthesis methods, water usage and treatment costs are expected to be comparable [4], emphasizing the importance of holistic environmental impact assessments. While hydrothermal and templating strategies may offer better alignment with green chemistry principles, their industrial applicability still requires validation at larger scales.

Ultimately, optimizing MXene synthesis requires a balanced consideration of technical feasibility, economic cost, and environmental sustainability. There remains an urgent need for systematic studies that compare the full environmental footprints of these methods under real-world scale-up conditions. Such efforts will be instrumental in guiding future decisions about which synthesis routes are best suited for commercial deployment in next-generation energy storage technologies.

## 10. Techno-Economic Discussion

The integration of MXenes as a next-generation alternative to graphite in industrial applications—particularly in energy storage—offers a compelling opportunity to advance both material performance and sustainability. Realizing this potential, however, depends on the development of cost-effective, scalable synthesis methods that can support large-volume manufacturing while maintaining the structural and functional integrity of MXenes. This technological challenge opens a vital pathway for innovation in synthesis science and industrial processing, encouraging the refinement of current techniques and the exploration of emerging methods.

While acid etching remains the most widely adopted approach due to its high yield of high-purity MXenes, its reliance on hazardous etchants such as hydrofluoric acid (HF) imposes significant safety, environmental, and cost burdens. As MXenes were first discovered via HF-based etching, this method has benefitted from early and extensive optimization. However, alternative techniques such as molten salt etching have gained momentum as safer and more sustainable solutions, replacing corrosive liquid acids with solid-state metal halides that offer improved handling and potentially lower environmental impact.

Vapor-phase deposition techniques, including chemical vapor deposition (CVD) and plasma-enhanced pulsed laser deposition (PEPLD), have demonstrated the ability to produce highly crystalline, uniform MXene films. These methods are particularly suited for applications requiring thin-film architectures, such as transparent conductors, supercapacitor coatings, or sensors. Nevertheless, their limited throughput and high equipment costs constrain their use for bulk MXene production, making them less suitable for applications like battery electrodes that require high material volumes.

In contrast, hydrothermal synthesis and template-assisted methods offer promising routes for enhanced production scalability. These bottom-up methods are generally simpler, involve fewer processing steps, and allow for the use of widely available metal salts and carbon/nitrogen sources. Although they may not always produce MXenes with the same crystallinity or purity as those obtained via acid etching, the resulting materials have shown sufficient quality for use in energy storage systems [65,66,67,68,69]. Moreover, these techniques align more closely with green chemistry principles and may prove more compatible with large-scale industrial manufacturing if process conditions are appropriately controlled.

Table 2 provides a comparative overview of large-scale production for both graphite and MXenes, analyzing key techno-economic parameters. The comparative analysis is structured around three principal criteria:

1. Precursor materials and their market availability and cost;
2. Operational expenses associated with each synthesis route (e.g., energy input, waste management, equipment complexity);
3. Material yield per unit of time or per reactor batch.

This assessment seeks to identify the most promising synthesis pathways for scaling up MXene production while maintaining economic feasibility. The research community is urged to direct efforts toward optimizing these production methods, reducing reliance on costly or hazardous inputs, and validating the performance of MXenes produced through alternative means. Only through such focused development will MXenes become viable for widespread integration into energy storage technologies, flexible electronics, and other emerging industrial applications.

## 11. Comparison of Methodologies for MXene Manufacturing

The scalability and sustainability of MXene production are deeply influenced by the chemical requirements, operational steps, and waste management protocols associated with each synthesis route. This section compares the key methodologies, focusing on precursor costs, safety, and environmental impact in the context of large-scale implementation.

Acid etching, the most established method for producing MXenes, raises significant concerns when considered for industrial-scale deployment. While the primary precursors—transition metals (M), group A elements, and carbon/nitrogen sources—are generally stable and non-hazardous in their solid, pelletized forms, the etchants used (e.g., hydrofluoric acid, hydrochloric acid, or strong bases) pose substantial challenges. These include handling hazards, toxicity, and the production of corrosive waste byproducts, both pre- and post-etching [70]. Scaling this method to ton-scale production would require large volumes of these hazardous chemicals, compounding both environmental and occupational safety risks.

Molten salt etching was developed as a safer alternative, substituting liquid etchants with solid-state halide salts (e.g., chlorides or fluorides) that are more stable at room temperature and present reduced handling risks. Post-etch waste from chloride-based salts is typically less toxic than that from acid etching, although not necessarily lower in volume. Moreover, this method introduces an additional high-temperature annealing step, increasing energy input and process complexity compared to room-temperature HF etching.

**Table 2:** Graphite vs. MXene synthesis and cost comparisons.

Material Source	Pros	Cons	Ideal Application	Material Cost	Operational Cost	Expected Scalability
Mineral graphite	Naturally abundant mineral	Finite mineral resource, not all of which is of battery quality	Low-end energy storage	$ (dependent on mineral licensing cost)	$ (Lump 2800 USD/ton flake 700–800 USD/ton) [71]	--
Mineral graphite (Processing Steps Example)	**Mining**			**Powder Milling**		
	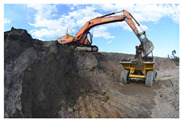			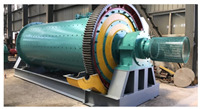		
**Material Source**	**Pros**	**Cons**	**Ideal Application**	**Material Cost**	**Operational Cost**	**Expected Scalability**
Synthetic graphite (MCMB, etc.)	Could be sourced from renewable precursors, and carbon sources are abundant and available	Usually sourced from petrochemicals and requires additional processing with high-temperature requirements (1700 °C)	Current industry standard for Li^+^ ion storage	$ (dependent on carbon source used, functionally free biomass waste could be used)	$$ (97–98% purity of Asia production 950–1450 USD/ton and 99.95% purity of Swiss production 7000–20,000 USD/ton) [71]	+
Synthetic graphite (Processing Steps Example)	**Graphitization**			**Powder Milling**		
	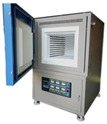			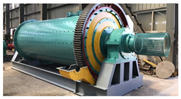		
**Material Source**	**Pros**	**Cons**	**Ideal Application**	**Material Cost**	**Operational Cost**	**Expected Scalability**
MXene from MAX Etch (Acid Etch)	Recipes exist for at least 24 different MXene types. The process produces high-quality and pure materials compared to other methods	The process uses harsh toxic/corrosive acids, which creates expensive waste to dispose of. Acid etch also has high temperature requirements (1000 °C+) and requires more equipment than other processes for much less material output	The current industry standard for MXene production, regardless of application, however, is that production volume is low	$$$ (estimated 12.3 million USD/ton) [72]	$$$ (estimated 20.33 million USD/ton) [72]	-
MXene from MAX Etch (Processing Steps Example)	**Melt Synthesis**	**Powder Milling**		**Acid Etching and Post-Etch Cleaning**		**Delamination**
	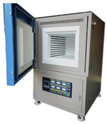	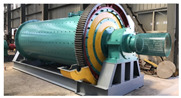		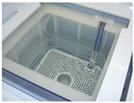		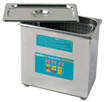
**Material Source**	**Pros**	**Cons**	**Ideal Application**	**Material Cost**	**Operational Cost**	**Expected Scalability**
CVD MXene	The process can produce high-purity, 2D semiconductor-quality materials	Material is produced in very small quantities, unsuitable for energy storage. The process also requires specialized equipment	Semiconductor applications such as transparent electrodes on LEDs, PVs	$$$ * (requires similar materials acid-etch)	$$ * (requires fewer steps than acid-etch per each batch)	+
CVD MXene (Processing Steps Example)	**CVD Growth**			**Delamination**		
	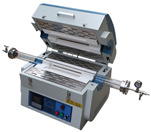			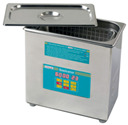		
**Material Source**	**Pros**	**Cons**	**Ideal Application**	**Material Cost**	**Operational Cost**	**Expected Scalability**
Hydrothermal MXene	The process can be scaled to produce large volumes of battery-quality material with recyclable waste products as well as lower temperature requirements (500–700 °C)	The process produces MXene composites, which would need further processing to increase the purity of the material. Material produced is limited to mostly molybdenum MXenes at this time	Replacement as electrode materials for Li^+^ ion storage	$$ * (uses cheaper materials than acid-etch)	$ * (requires fewer steps than acid-etch per each batch)	++
Hydrothermal MXene (Processing Steps Example)	**Hydrothermal Precipitation**	**Precipitate Washing**		**Annealing**		**Powder Milling**
	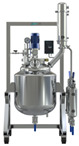	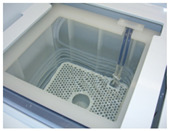		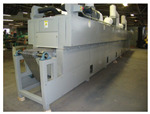		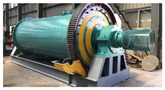

$—low cost, $$—medium cost, $$$—high cost, - unlikely, -- highly unlikely, + likely, ++ highly likely. * Cost estimates are comparing the materials used and process steps to the acid etch method.

In contrast, direct synthesis techniques such as chemical vapor deposition (CVD) and plasma-enhanced pulsed laser deposition (PEPLD) eliminate the need for corrosive etchants entirely. These bottom-up methods allow for in situ formation of MXenes on epitaxial substrates in a single-step process. CVD, in particular, can be scaled using standard tube furnaces, making it a more accessible option for industrial adoption. PEPLD, while capable of utilizing a broader range of precursors, requires specialized high-vacuum systems, which may constrain scalability due to high capital and operational costs. Both methods significantly reduce chemical waste compared to top-down etching approaches.

Hydrothermal synthesis has gained attention for its versatility across a wide range of applications, including energy storage [73], electrocatalysis [74], pesticide and organic contaminant removal [75,76], lubrication [77], and microwave absorption [78]. This method relies on relatively benign reagents such as water, biomass-derived carbon sources (e.g., sugars, starches), and transition metal salts. While some waste is generated during processing, it is generally easier and less expensive to manage than acid-based byproducts. For example, wastewater containing salts (e.g., ammonia, sodium, nitrates) can be treated more safely and economically. Moreover, the high-temperature annealing step often burns off residuals, further simplifying post-synthesis purification. Nonetheless, additional research is needed to fully evaluate the method’s scalability and long-term feasibility in industrial contexts.

Similarly, the templating method employs transition metal oxide precursors and carbon or nitrogen sources, but it also requires hydrogen and inert gases (e.g., argon) during the annealing process to facilitate the reduction of metal oxides into carbides or nitrides. While it shares the hydrothermal method’s advantages in terms of relatively benign precursors, the inclusion of high-temperature gaseous reduction steps introduces specific safety and equipment considerations. However, much of the process waste—such as carbon monoxide and volatile organic byproducts—is volatilized and captured during annealing. Furthermore, sacrificial template materials can often be reclaimed and reused, enhancing the method’s sustainability profile.

In summary, while all methods present trade-offs between material quality, scalability, and environmental burden, direct synthesis routes (e.g., CVD and hydrothermal) and molten salt methods offer notable advantages in terms of safety and sustainability compared to conventional acid etching. Continued development and optimization of these techniques, particularly in waste reduction and process efficiency, will be essential to enabling commercial-scale production of MXenes with minimal environmental impact.

## 12. Operational Requirements

When considering the replacement of graphite with MXenes in energy storage applications, it is essential to evaluate the operational requirements associated with MXene production. These include critical aspects such as energy consumption, equipment usage, and, most importantly, waste management. Among these, the cost and complexity of waste disposal often emerge as the most significant contributors to overall production expenses.

In the synthesis of MXenes—particularly through top-down routes involving the fabrication of MAX phase precursors—high-temperature furnaces play a central role. The energy demand and associated costs increase substantially with elevated processing temperatures (often reaching 1200–1600 °C) required for solid-state reactions. Additionally, the number of cycles and the duration of each thermal treatment directly influence operating costs. Further operational burdens arise when synthesis requires controlled atmospheres, such as hydrogen, inert gases (e.g., argon), or vacuum environments. These requirements introduce engineering and safety challenges at larger production scales, posing barriers to commercialization.

Ball milling, often employed for precursor homogenization or exfoliation steps, presents additional operational considerations. The milling duration, number of cycles, and power consumption must be optimized to reduce energy use without compromising material quality. However, these equipment-related costs are often overshadowed by the waste management burden, particularly when corrosive or toxic chemical reagents are used.

For example, acid-based etching techniques result in significant volumes of hazardous liquid waste, which require neutralization and safe disposal. This process entails substantial time, labor, and chemical input, often involving multistep treatment protocols. In comparison, wastewater containing ammonia or salt byproducts—common in hydrothermal methods—is far less hazardous and less costly to treat [79]. These distinctions make waste management a primary factor in determining the economic feasibility of MXene production.

The economic implications of these operational challenges are reflected in the market pricing of research-grade MXenes. Commercially available MXenes are priced between USD 192 and USD 900 per gram, depending on characteristics such as layer count and functionalization, as reported by suppliers including Sigma-Aldrich (Burlington, 01803 MA, USA) [80], 2D Semiconductors USA [81], and Thermo Fisher Scientific (Waltham, 02451, MA, USA). By contrast, the literature estimates suggest a theoretical production cost of approximately USD 20.33 per gram, based on lab-scale protocols [72]. This discrepancy is primarily due to the high cost of producing, storing, and shipping small research-scale quantities, as well as inefficiencies in batch processing and hazardous waste handling.

Within the estimated USD 20.33/g cost, Zaed et al. [72] report that the etching reagents—hydrochloric acid (HCl) and lithium fluoride (LiF)—alone account for USD 5.30 per gram, underscoring the financial weight of consumables in etching-based processes. As MXene production scales up, addressing these waste-related cost drivers becomes critical for ensuring economic viability and environmental sustainability.

Alternative synthesis strategies, such as hydrothermal [68] and templating methods, offer potential pathways for reducing waste and associated costs. These methods generally utilize benign reagents, avoid highly corrosive etchants, and may yield waste streams that are easier and cheaper to process. Consequently, further research and process development in these areas could play a pivotal role in enabling scalable, cost-effective MXene production, aligned with the demands of commercial energy storage applications.

## 13. MXene Products and Environmental Considerations

Product and byproduct management is intrinsically linked to the operational requirements of MXene synthesis and is a key factor in assessing the scalability and sustainability of production methods. While acid etching remains the most established synthesis route, its scale-up for industrial production is technically feasible given adequate infrastructure, equipment, and workforce. However, the significant waste generation, particularly involving corrosive byproducts, poses environmental and economic challenges. Innovative strategies, such as the recycling of etchants and water reuse protocols, have the potential to mitigate these impacts and reduce operational costs, aligning production with sustainability goals.

A notable concern in acid etching is the large volume of water required to dilute and process waste salts. Implementing closed-loop water systems for filtration and reuse can support both resource conservation and regulatory compliance, making large-scale acid-based MXene production more environmentally manageable.

In contrast, chemical vapor deposition (CVD) and plasma-enhanced pulsed laser deposition (PEPLD) techniques face constraints primarily due to low material yield relative to substrate area and the generation of inert solid waste. Although recycling of substrates has been explored [82,83], comprehensive strategies for integrating this into high-throughput systems are still lacking. Despite their precision and ability to yield highly crystalline films, the scalability of these techniques for bulk MXene production remains limited.

Hydrothermal and templating methods offer more promising pathways for sustainable scale-up. These approaches typically involve fewer hazardous reagents, generate lower volumes of toxic waste, and do not rely on strong acids or solvents [52,84]. In hydrothermal synthesis, the primary byproduct is ammonia-contaminated water, which is significantly less hazardous and more cost-effective to treat compared to acid wastes. As such, hydrothermal processes are well-positioned as more environmentally responsible alternatives for large-scale MXene synthesis.

Nevertheless, hydrothermal production at scale presents operational challenges that require careful engineering controls. Working with high-pressure stainless-steel autoclaves necessitates strict temperature regulation, pressure vessel integrity, and robust safety protocols to mitigate risks. While ammonia contamination is less severe than acid effluents, it still demands controlled disposal and proper wastewater treatment to ensure environmental compliance. Similarly, templating methods rely on the thermal reduction in metal oxides in the presence of hydrogen and inert gases, and while they involve manageable waste streams, the process still requires attention to volatile byproduct management and template reuse strategies.

To evaluate the long-term feasibility and sustainability of MXene production, a comprehensive life cycle assessment (LCA) is essential. To date, LCAs have been conducted primarily for Ti_3_C_2_T_x_ MXenes, particularly for production via selective acid etching [85]. While informative, these assessments must be expanded to include bottom-up synthesis routes, such as hydrothermal, CVD, PEPLD, and templating techniques. Such studies should account for critical metrics including reagent consumption, energy inputs, emissions, and waste handling costs across all stages of production.

A holistic LCA provides the framework to identify environmental bottlenecks, assess cost-effectiveness, and guide material selection and process optimization. Through this lens, researchers and manufacturers can make informed decisions that balance performance with sustainability, ensuring that MXene production technologies evolve in alignment with global environmental and industrial standards.

## 14. Additional Considerations and the MXene Marketplace

To further evaluate the commercial viability of MXenes in energy storage applications, a preliminary cost analysis was conducted based on the previously discussed synthesis methodologies. This evaluation was benchmarked against graphite-based lithium-ion battery systems, the current industry standard. The resulting comparison provides insights into the economic feasibility and scalability potential of MXene-based technologies in relation to the mature graphite supply chain. A summary of material, operational, and scalability considerations is provided in Table 2.

The cost structure associated with each synthesis route includes expenditures on core infrastructure—such as industrial furnace systems, ball milling equipment, and method-specific apparatus like hydrothermal autoclaves, sonication baths, and wash stations. Once a manufacturing facility is established, recurring material and energy costs, along with process optimization opportunities, can be evaluated to align the production costs of MXenes more closely with those of commercial graphite—without compromising the performance advantages of MXenes.

It is important to contextualize this comparison within the technology readiness levels of each material. Graphite, as a widely commercialized material, benefits from a mature global supply chain and widespread vendor availability. In contrast, MXenes remain largely confined to laboratory-scale synthesis, with only a handful of research-grade suppliers, such as Sigma-Aldrich, Thermo Fisher, and 2D Semiconductors USA, offering material at retail prices ranging from USD 192 to USD 900 per gram. These high costs reflect limited production volumes and the absence of large-scale manufacturing infrastructure.

Despite this disparity, MXenes exhibit promising market potential. According to Fortune Business Insights, the global graphite market is projected to grow from USD 8.32 billion in 2025 to USD 13.35 billion by 2032, at a compound annual growth rate (CAGR) of 6.9% [86]. In contrast, Industry ARC reports that the global MXene market—valued at USD 26.4 million in 2022—is forecast to reach USD 121.5 million by 2027, expanding at a CAGR of 29.24% over the 2022–2027 period [87]. These figures suggest strong projected demand growth, driven by MXenes’ multifunctional capabilities and emerging use cases in energy storage, electronics, and catalysis.

As MXene production scales and synthesis methods mature, material costs are expected to decline due to economies of scale, process optimization, and the establishment of dedicated manufacturing facilities. However, the timeline for widespread commercial adoption remains uncertain, contingent upon continued research and industry investment.

A key advantage of MXenes over graphite lies in their higher theoretical specific and volumetric capacities [88]. This opens opportunities to either reduce the active material mass for a given energy output or increase the energy density of a battery at a constant size. For instance, a Tesla Powerwall uses approximately 45 kg of graphite to deliver 13.5 kWh of energy [1]. If MXenes were to replace graphite as the anode material—assuming practical performance approaches their theoretical capacities (see Table 3 for detailed values)—the same energy output could potentially be achieved with a significantly smaller mass of active material. This capacity advantage could translate into lighter batteries, smaller form factors, or longer runtimes, offering flexibility across consumer and industrial energy storage applications.

In summary, while MXenes currently face economic and technical barriers to widespread commercialization, their superior performance metrics, rapid market growth, and process innovation potential position them as strong candidates for future energy storage technologies. Ongoing cost reductions and advances in synthesis will be pivotal in determining the trajectory of MXenes in competing with graphite and other incumbent materials in the marketplace.

## 15. Elemental Abundance of MXenes and Implications for Scalability

The scalability of MXene production for energy storage and other industrial applications hinges on the natural abundance, accessibility, and sustainability of its constituent elements. MXenes are primarily composed of transition metals combined with carbon, nitrogen, or both [4], offering a broad compositional space and tunable properties. However, the economic viability and environmental impact of large-scale MXene manufacturing are heavily influenced by the availability and extraction cost of these elements.

**Figure 2:**
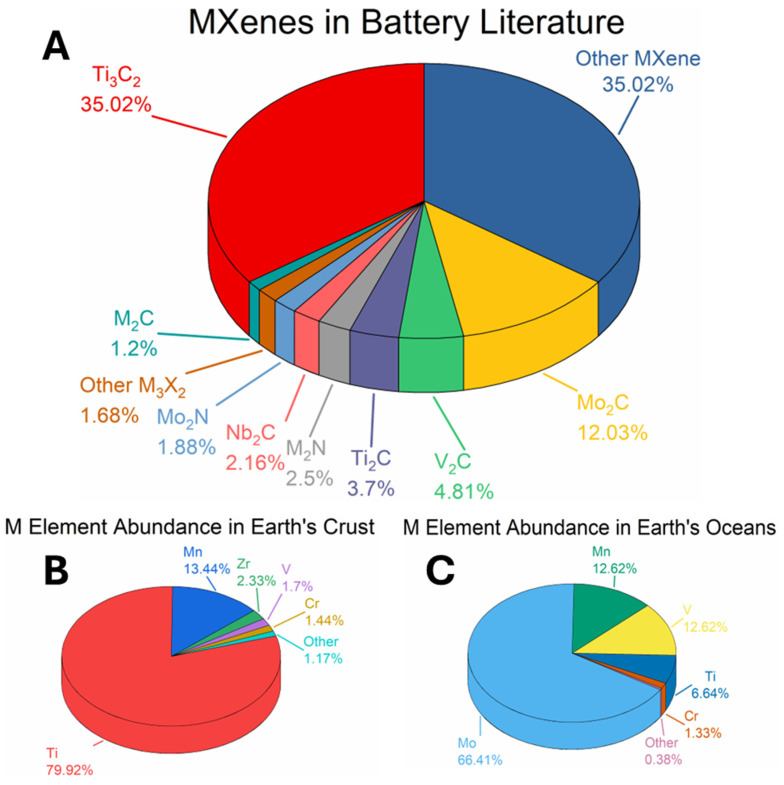
MXenes in battery research and natural elemental abundance of M-group metals. (**A**) Pie chart showing the distribution of MXene subtypes, based on keyword frequency in the Web of Science database. (**B**) Pie chart depicting the relative abundance of the 12 M-group elements in MXenes, according to their natural occurrence in the Earth’s crust (data from [89]) (**C**) Pie chart illustrating the elemental composition of oceanic seawater, a proposed resource for extracting lithium and other highly water-soluble minerals (data from [89]).

As shown in Figure 2, five transition metals—titanium (Ti), vanadium (V), chromium (Cr), manganese (Mn), and zirconium (Zr)—are among the most abundant and accessible in the Earth’s crust [89]. Of these, titanium is the most naturally abundant, which has contributed to the prevalence of Ti-based MXenes in research and development. These elements are routinely mined through well-established terrestrial extraction routes, making them relatively cost-effective and scalable for industrial use.

**Table 3:** Ab initio MXene (usually bare or unterminated) anode capacity.

MXene Material	Material Density (g/cm^3^)	Theoretical Gravimetric Capacity Li^+^ Ion (mAh/g)	Theoretical Volumetric Capacity of Li^+^ Ion (mAh/cm^3^)	Theoretical Gravimetric Capacity of Na^+^ Ion (mAh/g)	Theoretical Gravimetric Capacity of Al^3+^ Ion (mAh/g)
Graphite (graphene)	2.26 [90]	372 [21] (744)	840.7	—	—
Sc_2_C (monolayer)	3.11	462 ^a^ [14], (526 [7])	1635	362 ^a^ [14]	—
Ti_2_C (sheet)	4.52	430 ^a^ [11], (497 [7])	2112	344 ^a^ [11]	972 ^a^ [11]
V_2_C (monolayer)	5.74 [91]	411 ^a^ [11], (1412.39 [7])	2557	331 ^a^ [11]	941 ^a^ [11]
Cr_2_C	6.63	462 [7]	3063	—	—
Mn_2_C (monolayer)	—	(879 [92])	—	—	—
Y_2_C (monolayer)	4.63 [93]	—	—	(564 [12])	—
Zr_2_C	—	276 [7]	—	—	—
Nb_2_C (monolayer)	7.80 [94]	247 ^a^ [11], 271 [7] (542 [95])	2114	216 ^a^ [11], (271 [95])	623 ^a^ [11], (0 [95])
Mo_2_C (monolayer)	9.16 [96]	263 [7], (526 [97])	2409	(132 [97])	—
Hf_2_C	—	145 [7]	—	—	—
Ta_2_C	14.96	143 [7]	2139	—	—
W_2_C	17.17 [98]	141 [7]	2421	—	—
Sc_3_C_2_	—	337 [7]	—	—	—
Ti_3_C_2_	4.54 [99]	287 ^a^ [11], 320 [7]	1452	246 ^a^ [11]	710 ^a^ [11]
V_3_C_2_ (monolayer)	5.62	303 [7] (606 [13])	1702	(606 [13])	—
Cr_3_C_2_	—	298 ^a^ [7]	—	—	—
Zr_3_C_2_	—	180 ^a^ [7]	—	—	—
Nb_3_C_2_	—	177 ^a^ [7]	—	—	—
Mo_3_C_2_	—	172 ^a^ [7]	—	—	—
Hf_3_C_2_	—	96 ^a^ [7]	—	—	—
Ta_3_C_2_	—	95 ^a^ [7]	—	—	—
W_3_C_2_	—	93 ^a^ [7]	—	—	—
V_4_C_3_	—	223 [100]	—	223 [100]	—
Sc_2_N (monolayer)	—	(1547 [101])	—	—	–-
Ti_2_N (monolayer)	—	(484 [102])	—	(484 [102])	(45 [102])
Ti_2_NO_2_ (monolayer)	—	(378 [102])	—	(378 [102])	(1134 [102])
Y_2_N (monolayer)	—	(279 [101])	—	—	—

^a^ One source of variation in MXene capacity in ab initio data is whether or not the mass of the intercalated ion is included in the weight of the material for the gravimetric capacity values. The marked data values indicate cases where the article included the intercalated ion in the capacity per weight number, while the unmarked values do not.

In addition to terrestrial sources, oceanic brine and seawater are gaining recognition as alternative reservoirs for critical minerals, including those used in MXenes and battery technologies. For instance, while terrestrial lithium reserves are estimated at approximately 13 million tons, oceanic saltwater contains an estimated 231 billion tons, underscoring its vast and largely untapped potential [103,104]. Similarly, several MXene-relevant metals—such as titanium, vanadium, chromium, and manganese—are also present in oceanic environments in extractable concentrations.

A particularly promising element in this context is molybdenum (Mo). Though its terrestrial reserves are relatively limited (~11 million tons), it is the most abundant transition metal in ocean water, with estimated concentrations reaching 13 billion tons [104]. Molybdenum’s molybdate salts are highly soluble in water, making it a viable target for extraction from desalination reject brine, similar to emerging strategies for lithium recovery [105]. These factors position molybdenum as a sustainable and scalable candidate for MXene synthesis, particularly in regions investing in seawater desalination technologies.

The relationship between elemental abundance and research activity is also evident in the distribution of MXene studies. Titanium-based MXenes remain the most extensively investigated, reflecting their high natural abundance and processability. Molybdenum-based MXenes are gaining increasing attention, aligned with the element’s availability in oceanic reserves and its favorable electrochemical properties.

In summary, the future scalability of MXene technologies is strongly tied to the geochemical distribution of the transition metals involved. Prioritizing elements that are both abundant and sustainably sourced—from terrestrial or oceanic environments—will be essential to supporting the widespread deployment of MXenes in energy storage, catalysis, and beyond.

## 16. The Anode Capacity: Ab Initio Predictions for MXenes and Their Influence on Desired Material Properties

As with many crystalline materials, ab initio calculations offer a powerful tool for predicting the structural and electrochemical properties of MXenes prior to experimental synthesis. These first-principles simulations provide insights into trends in capacity, ion intercalation behavior, and surface reactivity, which can significantly guide the selection of promising MXene candidates for energy storage applications. A consolidated overview of such computational predictions is presented in Table 3, highlighting key structure–property relationships relevant to Li-ion and alternative ion systems.

One of the most striking trends in Li-ion battery systems is the dependence of gravimetric capacity on MXene layer architecture. While MXenes are generally denoted by the formula M_n+1_X_n_, where n = 1 or 2 (corresponding to M_2_X and M_3_X_2_ structures, respectively), M_2_X-type MXenes tend to offer higher gravimetric capacities due to their lower mass per formula unit. For example, theoretical calculations show that the Li-ion capacity of Ti_3_C_2_ (M_3_X_2_) is approximately 55% lower by weight than that of Ti_2_C (M_2_X), solely due to the increased atomic mass of the additional transition metal layer.

Furthermore, comparative studies suggest that MXenes derived from transition metals in the same period of the periodic table (e.g., Ti and Cr) display similar capacities. For instance, Ti_2_C exhibits only a 7% higher predicted Li capacity than Cr_2_C. However, when comparing elements within the same group, significant differences can emerge—W_2_C, for example, shows only 30% of the Li capacity of Cr_2_C [106]. These results underscore the importance of considering both electronic configuration and atomic mass when selecting M elements for optimal electrochemical performance.

Another critical consideration is the difference between monolayer and bulk MXenes. Much like the relationship between graphene and graphite, exfoliated monolayers of MXenes expose both top and bottom surfaces for ion intercalation, resulting in significantly higher predicted capacities. In the case of Li-ion intercalation, monolayer MXenes consistently show double the theoretical capacity of their multilayer or bulk counterparts. This is attributed to the additional intercalation sites available on both surfaces of the monolayer, as opposed to just the interlayer galleries in bulk structures.

However, it is important to recognize that not all MXenes outside the M_2_X architecture should be disregarded. Many M_2_X and M_3_X_2_ structures, especially those based on Ti, V, and Cr, exhibit superior volumetric capacities and molar energy densities compared to conventional graphite. This makes MXenes particularly attractive for applications where volumetric energy density is prioritized over gravimetric metrics—such as grid-scale storage, uninterruptible power systems (UPS), and stationary backup power. In these contexts, the higher density of MXenes enables more compact energy storage solutions, enhancing both operational efficiency and cost-effectiveness.

Beyond lithium-ion batteries, ab initio simulations have also identified MXenes as viable anode candidates for non-lithium-based ion systems, including sodium (Na^+^), potassium (K^+^), magnesium (Mg^2+^), calcium (Ca^2+^), and aluminum (Al^3+^) ions [11,107]. These systems are of growing interest due to the greater elemental abundance and lower cost of these ions compared to lithium. Importantly, many non-lithium systems suffer from poor compatibility with graphite and graphene, often due to weak ion intercalation or poor cycle life. In contrast, MXenes have demonstrated more favorable intercalation behaviors in computational studies and are further advantaged by their surface-terminating functional groups, which can be engineered to optimize ion adsorption and diffusion.

However, the capacity trends observed in lithium systems do not always translate directly to non-lithium ions. For instance, while monolayer exfoliation in lithium systems typically results in a doubling of capacity, this benefit is diminished or even negated for larger ions such as Na^+^ or multivalent species. In monolayer MXenes, high surface charge densities can lead to ion exclusion zones, reducing packing density and limiting intercalation. In contrast, bulk MXenes can stabilize these larger ions more effectively due to greater interlayer spacing and favorable electrostatics.

A representative example is Nb_2_C; when used as a Li-ion anode, the monolayer form exhibits nearly double the theoretical capacity of its bulk counterpart. However, in Na-ion systems, the monolayer version shows only an 18% increase, illustrating the non-uniform benefit of exfoliation across different ion chemistries. These insights emphasize the need for tailored MXene selection and structural design depending on the intended battery chemistry.

In conclusion, ab initio studies strongly support the promise of MXenes as next-generation anode materials, offering high gravimetric and volumetric capacities, tunable properties, and compatibility with a wide range of charge carriers. The challenge now lies in translating theoretical predictions into experimental validation, particularly through the development of scalable synthesis methods that preserve the structural and surface features critical to electrochemical performance.

## 17. Selected Electrochemical Storage Performance Data

The majority of experimentally validated electrochemical data available for MXenes comes from a relatively narrow subset of compositions, predominantly titanium-based (Ti) and molybdenum-based (Mo) MXenes. Titanium, the most abundant M-group transition metal in the Earth’s crust, is heavily represented in the battery literature. Both Ti_3_C_2_ and Ti_2_C appear extensively in experimental studies, reflecting their accessibility, structural stability, and established synthesis routes.

Molybdenum-based MXenes, particularly Mo_2_C and Mo_2_N, are also well-represented in the literature. This interest is driven not only by their favorable electrochemical properties but also by the abundance of molybdenum in oceanic reserves, where its high water solubility supports potential extraction from desalination brine streams. A Web of Science survey of peer-reviewed publications confirms the dominance of Ti- and Mo-based systems in experimental MXene battery research.

It is important to note that not all MXenes are viable candidates for replacing graphite in lithium-ion battery anodes. However, experimental data compiled in Table 4 highlights several MXenes that exceed the theoretical gravimetric capacity of graphite (372 mAh/g)—even at early stages of development. These findings underscore the significant promise of MXenes as next-generation anode materials, particularly for high-capacity energy storage systems.

**Table 4:** MXene experimental data.

Material	Anode or Cathode for Ion	Experimental Gravimetric Capacity (mAh/g)	Voltage Range Tested (V)	Cycle Rate (mA/g)	Refs.
Ti_2_C	Li anode	515.3	0–3	100	[108]
	Li anode	146.2	0–3	3000	[108]
V_2_C	Li anode	260	0–3	370	[109]
	Al cathode	31.5	0–2.5	1000	[110]
V_2_C treated with Se	Al cathode	119.8	0–2.5	1000	[110]
Nb_2_C	Al cathode	108	0.1–2.4	0.2	[111]
	Al cathode	80	0.1–2.4	0.5	[111]
Mo_2_C	Li anode	494	0–3	5	[41]
	Li anode	50	0–3	10	[41]
	Li anode	38	0–3	50	[41]
Mo_2_C/MoO_2_/C composite	Li anode	700	0–3	500	[66]
Ti_3_C_2_	Li anode	87.4	0–3	87.4	[112]
	Li anode	147.4	0–3	147.4	[113]
	Al cathode	455.5	0.4–2.4	100	[26]
Ti_3_C_2_ treated to terminate in sulfur	Na anode	135	0–2.5	2000	[114]
Ti_3_C_2_ treated to add antimony oxide	Na anode	365	0–2.5	4000	[115]
Ti_3_C_2_/S mix	Li cathode	1120	1.7–2.8	224	[116]
Ti_3_C_2_-VS_2_ composite mixed with sulfur	Li cathode	1212	1.7–2.8	242.4	[116]
Ti_3_C_2_ treated to add CTab-Se	Al cathode	583.7	0.4–2.4	100	[26]
Ti_3_CN	Na anode	103	(0–3)	20	[27]

The ability of some MXenes to deliver higher specific capacities than graphite, alongside their superior volumetric energy density and tunable surface chemistry, positions them as compelling alternatives for future commercialization and scalable battery manufacturing. Continued development of these high-performing compositions, coupled with advances in large-scale synthesis and integration into full-cell architectures, will be key to realizing their potential in practical applications.

## 18. Ti_3_C_2_T_x_ MXenes in Battery Energy Storage

Ti_3_C_2_T_x_, the first synthesized and most extensively characterized MXene, continues to lead research across the broader MXene family—particularly in the field of energy storage. As illustrated in Figure 2, Ti_3_C_2_T_x_ accounts for approximately 35% of all published MXene battery research, far surpassing the next most studied material, Mo_2_C, which comprises roughly 12%. This widespread focus reflects Ti_3_C_2_T_x_’s favorable electrochemical properties and synthetic accessibility.

Ti_3_C_2_T_x_ has been investigated for a wide range of battery chemistries, serving as an anode material in Li-ion and Na-ion batteries and as a cathode in Al-ion systems, as summarized in Table 5. These studies underscore the material’s versatility and reinforce its position as a benchmark MXene for electrochemical performance evaluation.

Surface terminations have a profound influence on the theoretical and experimental capacities of Ti_3_C_2_T_x_. Ab initio simulations have shown that fluorine-terminated surfaces can significantly reduce gravimetric capacity—from approximately 320 mAh/g [7] for pristine surfaces to 130 mAh/g [117] for fully fluorinated ones—due to the added mass of non-conductive terminations. This highlights the critical role of synthesis techniques, particularly the widely used HF-based etching, which often yields mixed or uncontrolled surface functional groups. Such non-ideal terminations can add parasitic mass without contributing to electrochemical activity, partially explaining why experimental capacities often fall short of theoretical predictions.

**Table 5:** Ti_3_C_2_ experimental and theoretical results side by side.

Application	Experimental Gravimetric Capacity (mAh/g)	Theoretical Gravimetric Capacity (mAh/g)	Percent Experiment Differs from Theory
Li-ion anode	147.4 [113]	287 ^a^ [11] 320 [7]	46%
Li-ion anode fluorine-terminated Ti_3_C_2_F_2_	123.6 [118]	130 [117]	4.9%
Na-ion anode	200 [119]	246 ^a^ [11]	16.7%
Na-ion anode sulfur-terminated Ti_3_C_2_S_x_	136.6 [114]	173.6	21%
Al-ion anode	—	~700 ^a^ [11]	—
Al-ion cathode AlCl_4_^−^ ion intercalation	455.5 [26]	—	—

^a^ One source of variation in MXene capacity in ab initio data is whether or not the mass of the intercalated ion is included in the weight of the material for the gravimetric capacity values. The marked data values indicate cases where the article included the intercalated ion in the capacity per weight number, while the unmarked values do not.

To address these limitations, refined manufacturing techniques that promote controlled and optimized surface terminations are essential. Enhancing surface chemistry is particularly important for applications where rate capability and cycling stability are key.

Early experimental studies of Ti_3_C_2_T_x_ MXenes have also revealed the material’s susceptibility to parasitic capacitance, a phenomenon attributed to their large surface area, high conductivity, and two-dimensional morphology—traits characteristic of supercapacitor electrodes. This dual functionality has positioned Ti_3_C_2_T_x_ as a strong candidate for hybrid applications bridging capacitive and battery-type storage.

For instance, Li et al. demonstrated that Ti_3_C_2_T_x_ (T = OH, O) synthesized via an alkali-assisted hydrothermal method retained 89.1% capacitance over 10,000 cycles at a current density of 5 A/g, showcasing excellent durability for supercapacitor applications [120]. Building on this, Ren et al. introduced a microemulsion system incorporating ionic liquids (ILs), which remain confined within the MXene interlayers post-annealing. This system functions both as an electrolyte and an interlayer spacer, dramatically increasing the accessible surface area and thereby enhancing charge storage performance [106].

These findings illustrate the flexibility of Ti_3_C_2_T_x_, not only as a battery electrode but also as a platform for electrochemical innovation. They also reflect how the research focus on a material can evolve as the community’s understanding deepens.

For example, while early ab initio studies suggested the potential for reversible aluminum-ion intercalation in MXenes—supporting their use as anodes—experimental efforts have predominantly shifted toward MXene cathodes in Al-ion batteries. This shift reflects the field’s broader goal of identifying stable Al-ion cathode materials to pair with metallic aluminum anodes, which are attractive due to their high capacity and dendrite-free behavior.

Ti_3_C_2_T_x_ has demonstrated promising performance as an Al-ion battery cathode, outperforming conventional hard carbon electrodes [26]. In these systems, Ti_3_C_2_T_x_ acts as an effective host for aluminum chloride complex ions, generated via the oxidation of the Al metal anode. This showcases the importance of aligning material properties with system-level requirements and how MXene research continues to evolve in response to technological needs.

## 19. Charge Storage Mechanisms and the Effect of Functional Groups

The potential of MXenes to replace graphite as the dominant electrode material has garnered considerable attention in recent years due to their tunable structure, surface chemistry, and superior electrochemical properties [121,122]. However, a major challenge in scaling up MXenes for industrial applications lies in the incomplete understanding of their charge storage mechanisms, particularly their ion intercalation/deintercalation behavior during cycling. Unlike conventional graphite, MXenes can assume various crystal structures and elemental combinations, and their surface functional groups—determined largely by the etching method used—significantly influence their electrochemical performance and storage mechanism.

To guide the rational design of high-performance MXenes and accelerate their commercialization, Table 6 summarizes the reported charge storage mechanisms for Ti_3_C_2_T_x_, the most studied MXene, across Li-ion, Na-ion, and Al-ion battery systems, highlighting the role of surface terminations in each case.

## 20. Li-Ion Systems: Surface Engineering for Zero-Strain Intercalation

In Li-ion batteries, graphite serves as the standard anode material. However, it suffers from structural degradation at high cycling rates due to up to 10% volumetric expansion during lithium-ion intercalation [123,124,125]. To overcome these limitations, Wang et al. investigated the lithium storage behavior of Ti_3_C_2_T_x_ and enhanced its capacity and cycling stability through ammoniation-based surface modification [126,127].

**Figure 3:**
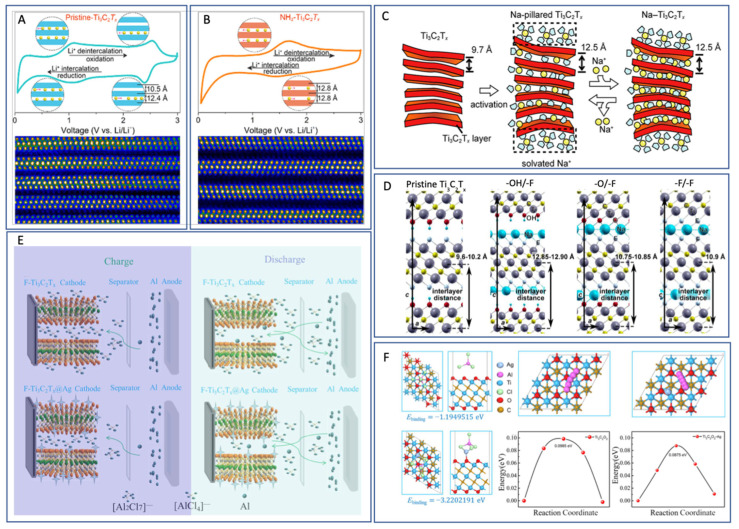
Charge storage mechanisms and the effect of functional groups of MXene-based ion batteries under discussion. (**A**) Cyclic voltammetry profile and Li^+^ (de)intercalation mechanism of the pristine-Ti_3_C_2_T_x_ electrode [126]. Copyright 2018, American Chemical Society. (**B**) Cyclic voltammetry profile and Li^+^ (de)intercalation mechanism of NH_3_-Ti_3_C_2_T_x_ electrode [126]. Copyright 2018, American Chemical Society. (**C**) Schematic illustration for the proposed mechanism of Na^+^ insertion into Ti_3_C_2_T_x_ [130]. Copyright 2016, American Chemical Society. (**D**) Optimized structures for pristine Ti_3_C_2_T_x_ and Na^+^-intercalated Ti_3_C_2_T_x_ with the various termination groups –OH/–F, –O/–F, and –F/–F [130]. Copyright 2016, American Chemical Society. (**E**) Charge/discharge mechanism models of F-Ti_3_C_2_T_x_@Ag and F-Ti_3_C_2_T_x_ in aluminum batteries [132]. Copyright 2021, American Chemical Society. (**F**) Adsorption of Ti_3_C_2_O_2_ and Ti_3_C_2_O_2_–Ag on AlCl_4_^−^; models of the AlCl_4_^−^ anions migrate between Ti_3_C_2_O_2_ and Ti_3_C_2_O_2_–Ag; barrier diagrams when the AlCl_4_^−^ anions are intercalated/de-intercalated between Ti_3_C_2_O_2_ and Ti_3_C_2_O_2_–Ag layers [132]. Copyright 2021, American Chemical Society.

Cyclic voltammetry (CV) data (Figure 3) for pristine Ti_3_C_2_T_x_ revealed two oxidation and two reduction peaks, corresponding to sequential Li^+^ intercalation into its interlayer spaces with d-spacings of 12.4 Å and 10.5 Å, respectively. STEM imaging confirmed non-uniform interlayer spacing in the pristine material, leading to a stepwise intercalation process. In contrast, NH_3_-functionalized Ti_3_C_2_T_x_ showed a uniform interlayer spacing of 12.8 Å and a single pair of redox peaks in CV, indicating simultaneous intercalation and deintercalation with a zero-strain behavior. This structural uniformity enables higher rate capabilities and improved capacity retention compared to unmodified Ti_3_C_2_T_x_.

## 21. Na-Ion Systems: Interlayer Flexibility and Solvent Effects

Sodium-ion batteries are emerging as an attractive alternative due to sodium’s low cost and natural abundance [128]. However, the larger ionic radius of Na^+^ makes graphite unsuitable as an anode [129]. MXenes, particularly Ti_3_C_2_T_x_, have demonstrated high capacity and stability due to their flexible interlayer spacing.

Yamada et al. examined Na^+^ intercalation in Ti_3_C_2_T_x_ nanosheets and observed an expansion of the interlayer spacing from 9.7 Å to 12.5 Å during the first intercalation cycle [130]. This expansion is attributed to the partial desolvation of Na^+^ and the subsequent insertion of uncoordinated solvent molecules into the interlayer, resulting in irreversible capacity loss in the first cycle. In subsequent cycles, the spacing remained stable at 12.5 Å, supported by Na^+^-solvent pillar structures. DFT calculations further show that surface functional groups affect interlayer spacing, with –OH terminations causing the most significant expansion due to strong repulsive interactions with sodium ions, potentially leading to distinct charge storage behavior.

**Table 6:** Ti_3_C_2_T_x_ charge storage mechanisms and effect of functional groups.

Application	Intercalated Ions and Radius	Functional Groups	Working Mechanisms	References
Li-ion anode	Li^+^, 0.76 Å	pristine	Lithium ions first intercalate into the larger interlayer spacing (12.4 Å) and then into the smaller interlayer spacing (10.5 Å), while the de-intercalation process is the opposite	[126]
Li-ion anode	Li^+^, 0.76 Å	-NH_3_	The interlayer spacing is uniform at 12.8 Å. This leads to simultaneous lithium-ion intercalation and deintercalation with a zero-strain feature	[126]
Na-ion anode	Na^+^, 1.06 Å	pristine/ -O/ -F	Expansion of interlayer distance during first sodiation (from 9.6 to 10.2 Å to 10.55–12.5 Å); interlayer distance does not change after activation due to pillared Na ions and swelling effect of solvent molecules	[130]
Na-ion anode	Na^+^, 1.06 Å	-OH	-OH greatly widens the interlayer spacing to 11.85 Å due to the strong repulsion between sodium ions and H atoms; before first sodiation, the interlayer spacing increases to 14.65 Å after sodiation	[130]
Al-ion cathode	AlCl_4_^−^, 2.64 Å	-F and -OH	Highly reduced Ti ions interact with AlCl_4_^−^ and lead to the incomplete removal of AlCl_4_^−^ ions during the charging and discharging process	[131,132]
Al-ion cathode	AlCl_4_^−^, 2.64 Å Al^3+^, 0.54 Å	-F, -OH and -Ag	The active Ti ions and functional groups (-OH, -F) are locked by tiny Ag particles, and Ag^+^ is reduced to Ag, effectively preventing the interaction between Ti_3_C_2_T_x_ and AlCl_4_^−^, and maintaining unobstructed migration channels for AlCl_4_^−^	[131,132]

## 22. Al-Ion Systems: Overcoming Interlayer Constraints Through Surface Modification

Aluminum-ion batteries typically use metallic aluminum as the anode, while graphite, with its narrow 5.7 Å interlayer spacing, struggles to accommodate the large **AlCl_4_^−^ anions (~5.28 Å)** [133]. MXenes, with tunable interlayer spacing, offer a compelling alternative cathode platform for Al-ion systems [134]. However, conventional etching methods introduce a high density of –OH and –F groups and reduced Ti species, which strongly interact with AlCl_4_^−^ and impede its removal during cycling—leading to irreversible capacity loss [130].

To address this, Huo et al. developed Ag^+^-modified Ti_3_C_2_T_x_ by incorporating ~100 nm silver nanoparticles, which chemically fix the reactive Ti centers and surface terminations [131,132]. As shown in Figure 3, this modification prevents unwanted AlCl_4_^−^ binding, maintains open migration channels, and enables efficient ion transport. The modified Ti_3_C_2_O_2_ demonstrated lower adsorption energy and reduced intercalation barriers, along with Coulombic efficiencies exceeding 100%, indicative of partial Al^3+^ intercalation due to improved ion accessibility. The result is a higher reversible capacity and enhanced cycling stability compared to unmodified Ti_3_C_2_T_x_.

MXenes exhibit ion-specific charge storage mechanisms that are highly dependent on surface terminations, interlayer spacing, and ion-solvent interactions. While pristine Ti_3_C_2_T_x_ displays stepwise intercalation behavior, surface-engineered MXenes can achieve simultaneous, zero-strain ion storage, improving rate performance and structural integrity. Moreover, their applicability extends beyond Li^+^ systems to Na^+^ and Al^3+^ chemistries, where graphite fails due to size constraints or poor compatibility.

The development of functional group-controlled MXenes and the integration of novel modification strategies such as metal ion doping will be essential for advancing their practical use. Understanding these mechanisms at both theoretical and experimental levels is critical to unlocking the full potential of MXenes in next-generation, high-performance, and sustainable battery technologies.

## 23. Case Studies of MXenes in Solid-State Batteries (SSBs)

### 23.1. MXene Composite Materials for Anodes

**Figure 4:**
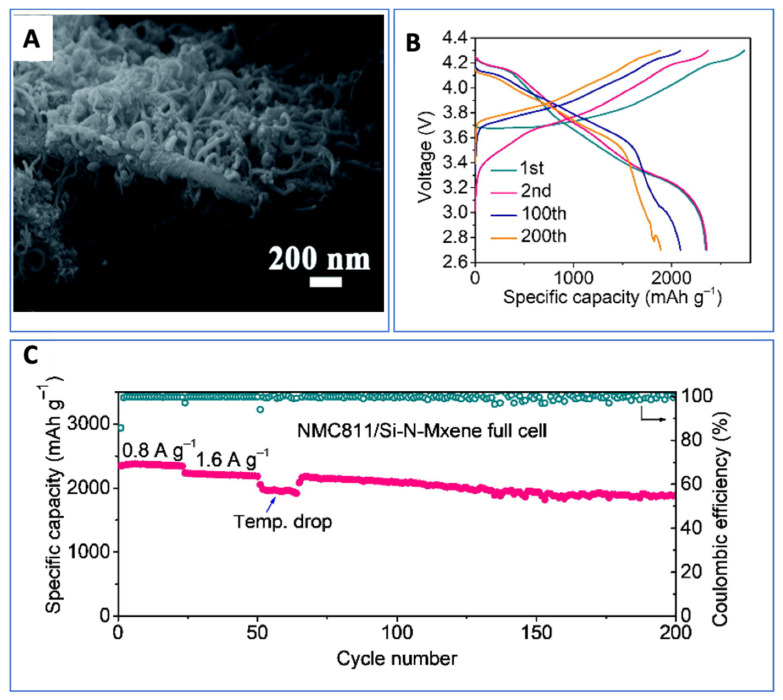
Characterization of MXene anode composites and electrochemical performance. (**A**) SEM image of the schematic of the CoS_2_/CNTs/TiO_x_N_y_ composite [135]. (**B**) Voltage profile of the full cell at different cycle numbers [137]. (**C**) The cycling performance of NMC811/Si-N-MXene full cell [137].

MXenes are incorporated into anode composites to enhance SSB performance, summarized in Table 7. For solid-state sodium-ion batteries (SSIBs), the scarcity of anode materials for selection is the major limiting factor of development. In situ growth of cobalt disulfide and carbon nanotubes on the MXene (Ti_3_C_2_T_x_) forms the CoS_2_/CNTs/TiO_x_N_y_ composite, exhibiting promising electrochemical properties. Mengli et al. combined the as-prepared anode with PFSA-Na membranes as the SSE for solid-state sodium-ion batteries (SSIB), as shown in Figure 4A [135].

**Table 7:** Comparison of anode/MXene composites for SSBs.

Anode Composite	Other Additives	Capacity (mAh g^−1^)	Cycle Life	Rate Capability	Notes	Refs.
CoS_2_/CNTs/TiO_x_N_y_/Ti_3_C_2_T_x_	NA	150	100	2000 mAh g^−1^ @	CoS_2_/CNT grown in situ on MXene	[135]
Li/Ti_3_C_2_T_x_	NA	140 (paired with LFP)	100	0.5 C	The composite reduces the interfacial resistance of Li/Garnet SSE from 1291 Ω cm^2^ to 5 Ω cm^2^	[136]
Si/N-doped Ti_3_C_2_T_x_	PAN	1888	200	1498 mAh g^−1^ @ 6400 mA	The nitrogen bond stabilizes the solid electrolyte interphase layer and facilitates Li^+^ transport	[137]
TiNb_2_O_7-x_/Ti_3_C_2_T_x_	NA	264	1000	5 C	Oxygen vacancy on the TNO promotes electronic conductivity by around four orders of magnitude	[138]

Solid-state electrodes (SSEs) are used to suppress their high shear modulus [139] to prevent lithium dendrite formation on the Li metal. However, the poor interfacial compatibility between Li metal and SSEs has bottlenecked the combination. MXene is introduced to Li metal to mitigate the challenge of forming Li/MXene composite, which decreases surface tension [136]. Ti_3_C_2_T_x_ is mixed with molten Li at 260 °C. The fluorine-terminated Ti_3_C_2_T_x_ forms LiF with Li during the mixing process. The reaction between Ti_3_C_2_T_x_ and Li metal was confirmed by XPS. The Ti-F peak shifted to 684.5 eV, indicating LiF formation from the reaction of Li metal with Mxene. The authors also confirm that a gradual decrease in LiF correlates with increasing depth, signifying its relatively higher concentration on the top surface. LiF’s negligible electron conductivity allows the in situ formed layer at the Li-MXene/garnet interface to effectively block SSE-Li side reactions and inhibit dendrite growth. LiF can also effectively suppress the formation of Li dendrite thanks to its high Young’s modulus and low conductivity [140].

Si anodes are promising candidates for next-generation solid-state lithium-ion batteries. Si anodes are known to undergo a drastic volume change during lithiation/delithiation, which limits their application [141]. Han et al. utilized MXene and PAN to synthesize a self-integrated Si/Ti_3_C_2_T_x_ anode composite with enhanced interfacial adhesion between Si and the MXene, stabilizing the composite structure, as shown in Figure 4C [137]. Initially, Si, PAN, and the MXene material were mixed in a DMF-based slurry with a mass ratio of 90:40:10. During the heating process, PAN transforms into amorphous carbon, providing nitrogen bonds with Si and the MXene. This unique bonding facilitates rapid Li^+^ and electron transportation. The amorphous carbon shields the Si particle to prevent volume change and consequent cracking of the Si. The full cell composed of NMC811 as the cathode and Si-N-Mxene as the anode was tested. Figure 4B shows the galvanostatic charge–discharge (GCD) profile of the cell. As the cycle number increases, plateaus move upwards of the charge curve, indicating increased cell total impedance. The discharge plateaus shortened after cycling, showing decreased reversible Li^+^ capacity. This could probably be due to the reduced lithiation sites caused by delaminated Mxene layers as Si particles continuously expand and contract over cycling. As shown in Figure 4C, the fabricated anode composite exhibited an initial discharge capacity of 2346 mAh g^−1^ at a high current of 1.6 A g^−1^, with an 80.5% capacity retention after 200 cycles.

### 23.2. MXene Composite Materials for Cathodes

**Figure 5:**
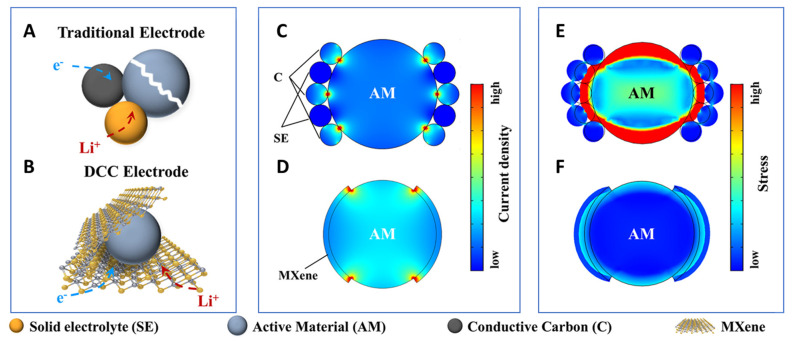
Schematics and simulations for traditional and DCC electrodes [143]. (**A**) Schematic of the traditional composite electrode and conduction pathways of electrons and Li^+^. (**B**) Schematic of the DCC electrode and the dual-conduction pathways of electron and Li^+^ in the Mxene. (**C**) Current density distribution simulation in a traditional composite electrode. (**D**) Current density distribution simulation in the DCC electrode. (**E**) Mechanical stress distribution simulation in a traditional composite electrode. (**F**) Mechanical stress distribution simulation in the DCC electrode.

In all-solid-state batteries, the solid–solid interfaces dominate the kinetics of charge transfer and diffusion. In a conventional composite electrode, the three phases of conductive additive, active material, and solid-state electrolyte introduce high complexity of interfacial challenges, including electrolyte decomposition, limited charge transfer rate, thermal instability, and mechanochemical coupling effects [142] (Figure 5A). Table 8 summarizes representative studies of the cathode/MXene composites for SSBs. A direct way to alleviate the challenges is to reduce the number of phases in the composite electrode. Therefore, fewer interfaces are presented. To achieve this, a dual-function component that can conduct ions and electrons is needed. An all-in-one ionic-electronic dual carrier conductor (DCC) provides the same diffusion channel for both Li^+^ and electrons. Fu et al. designed a dual-conductive cathode material with only the active material and MXene. The schematic figure is shown in Figure 5B [143]. Among all the mixtures of active materials and MXene, the Ge/Mxene composite showed the best performance. An aerial capacity of 6.72 cm^−2^ was achieved at the current density of 1.6 mA cm^−2^. The composite with only 10 wt% of MXene exhibited an excellent capacity of 615 mAh g^−1^ after 20 cycles. The simplicity of the electrode system resulted in reduced interfacial challenges and improved electrochemical performance. Figure 5C,D simulate the current density in the traditional and DCC electrodes. More evenly distributed current density can be seen in the MXene/active material composite. Similarly, as shown in Figure 5E,F the DCC electrode has less aggregated mechanical strength than the conventional electrode system.

**Table 8:** Comparison of cathode/MXene composites for SSBs.

Cathode Composite	Other Additives	Capacity (mAh g^−1^)	Rate Capability	Cycle Life	Note	Refs.
Ge/Ti_3_C_2_T_x_	NA	950	800 mAh g^−1^ @ 1.6 mA cm^−2^	70	MXene is utilized to provide a dual ionic–electronic transport network and control the volume expansion in the cathode	[143]
S/Ti_3_C_2_T_x_	COF-derived N-doped carbon	584	400 mAh g^−1^ @ 500 mA g^−1^	100	The CTT/MXene paper acts as an electronic conductive host for sulfur	[144]
Iodine/Ti_3_C_2_T_x_	Carbon cloth	300	200 mAh g^−1^ @ 10 C	1000	MXene-wrapped carbon cloth–iodine dispersed in CPE	[145]

Lithium-iodine (Li-I) batteries have garnered significant interest in recent times due to their high energy and power densities, coupled with the cost-effectiveness of iodine [145]. However, the shuttle effect of iodine and the uncontrolled growth of lithium dendrites have posed practical challenges to their usage. To address this issue, a quasi-solid-state Li-I battery was developed by utilizing an MXene-based iodine cathode and a composite polymer electrolyte (CPE) that incorporates NaNO_3_ particles in a pentaerythritol-tetraacrylate-based (PETEA-based) gel polymer electrolyte. This proposed solution represents a significant step in making Li-I batteries viable for practical applications [145]. The performance of Li-I batteries can be significantly enhanced by leveraging the abundant functional groups present on the surface of MXene sheets. These functional groups offer robust chemical binding capabilities to iodine, thereby limiting their shuttling and suppressing their diffusion. Additionally, the use of a PETEA-based polymer matrix effectively stabilizes the Li anode/CPE interface against dendrite growth [145]. Furthermore, the incorporation of NaNO_3_ particles serves as an effective catalyst for the transformation kinetics of LiI_3_ on the cathode. The combination of these synergistic optimizations results in superior energy/power density, long-term cycling stability, and excellent flexibility in lithium-ion batteries.

### 23.3. MXene Composite Materials for Solid-State Electrolytes (SSE)

Solid-state electrolytes (SSE) enable the practical design of Li-metal batteries (LMB) due to SSE’s high mechanical strength, which can suppress Li dendrite formation. To release the full performance of LMBs, challenges, including low ionic conductivity of SSE, interfacial instability and resistance, and brittleness of SSEs, need to be solved [146]. When used as a filler in solid polymer electrolyte (SPE), MXenes render reduced crystallinity of the polymer and increased ionic conductivity by enhancing the interaction with the polymer chains due to their large surface area and hydrophilic terminal groups. The incorporation of MXenes can also bolster mechanical strength, regulate ion transport, and hinder glass transition in solid-state electrolytes, thus advancing their practical applicability [147]. Table 9 demonstrates the latest studies on the incorporation of MXene in the SSEs.

**Table 9:** Comparison of SSE/MXene composites for SSBs.

Type of MXene	SSE Composite	Other Additives	Conductivity (S cm^−1^)	Notes	Refs.
Ti_3_C_2_T_x_	PEO/Ti_3_C_2_T_x_	LiTFSI	2.20 × 10^−5^	3.6 wt% MXene in the SPE composite reduces the crystallization of PEO and enhances the overall ionic conductivity	[148]
Ti_3_C_2_T_x_	PVHF/MXene-g-PMA	Zn(OTF)_2_	1.24 × 10^−5^	MXene grafted with PMA promotes the formation of hydrogen bonding with PVHF	[149]
Ti_3_C_2_T_x_	PVA-TiO_2_	Zn(CF_3_SO_3_)_2_	1.24 × 10^−5^	2D TiO_2_ nanosheets are fabricated from Ti_3_C_2_T_x_ through a hydrothermal reaction	[150]
Ti_3_C_2_T_x_	PH SPE/Ti_3_C_2_T_x_	NA	4.52 × 10^−4^	MXene-bonded transport network	[151]

The utilization of 2D materials for composite polymer electrolytes (CPEs) appears to hold great promise due to their higher specific surface areas than 0D or 1D materials. This unique 2D property enables anisotropic properties in CPEs. Notably, in-plane conductivity along the 2D filler surface can be two orders of magnitude higher than that along the normal direction of the filler [148]. With only 3.6 wt% MXene added into the PEO/LiTFSI CPE, the ionic conductivity increased threefold from 6.4 × 10^−6^ S cm^−1^ of the MXene-free sample to 2.2 × 10^−5^ S cm^−1^. The DCC property of MXene may raise concerns about increased electronic conductivity and consequent metallic Li formation in CPE. The electronic conductivity of the PEO/LiTFSI/MXene, however, still remains at the magnitude of 10^−10^ S cm^−1^.

**Figure 6:**
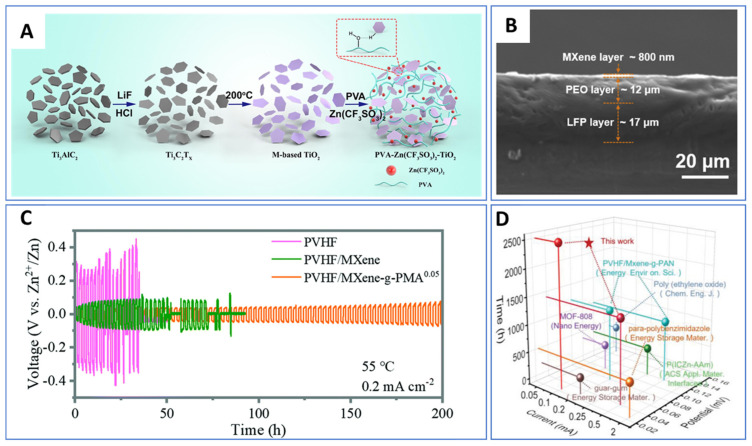
SSE/MXene composites for SSBs. (**A**) Schematic of the fabrication process of TiO2-based CPE from Ti3C2Tx MXene [150]. (**B**) Cross-sectional SEM image of MXene-PEO-LFP [152]. (**C**) Cycling test of Zn/Zn symmetrical cells based on the various SPEs at 55 °C [149] (**D**) Significant increase in cycle lifespan of the PH SPE/Ti_3_C_2_T_x_ SSE compared to other works [151].

To promote the dispersion of MXene in the SPE based on poly(vinylidene fluoride-co-hexafluoropropylene) (PVHF) for Zn-ion SSB, Chen et al. grafted Ti_3_C_2_T_x_ with poly(methyl acrylate). From Figure 6C, grafted MXene exhibited a significantly stabilized cycling performance, attributed to the high ionic conductivity and Zn^2+^ transference number.

MXenes can also be used as a precursor for fabricating 2D TiO_2_ nanosheets for CPE in a Zn-ion SSB [150]. As shown in Figure 6A, TiO_2_ nanosheets are formed by a one-step hydrothermal reaction following the synthesis of Ti_3_C_2_T_x_ MXene. After mixing with PVA and Zn(CF_3_SO_3_)_2_, the CPE is prepared for a full SSB cell. Zn dendrite formation is efficiently suppressed by the CPE containing only 3% of TiO_2_, which leads to superior stable cycling. With a high coulombic efficiency of 99.8% after 115 cycles, the full Zn metal cell paired with VO_5_ retained a high discharge capacity of 216 mAh g^−1^.

## 24. Incorporation of MXene Materials at the Electrode/Electrolyte Interface

To regulate Li deposition, researchers have been incorporating MXene laminates at the SPE/Li metal interface. The rigid MXene stacking layer can provide higher compressive strength than SPE, which helps inhibit Li dendrite formation. The relatively higher electronic conductivity compared to SPE promotes directional and local Li deposition. Meanwhile, the substantial amount of negatively charged groups attached to the MXene promotes even Li-ion distribution and, therefore, uniform Li deposition at the interface [153].

Weijie et al. introduced Ti_3_C_2_T_x_ at the PEO/Li metal interface by electrospinning a thin layer of the MXene/PEO/LiTFSI composite (Table 10) [152]. From Figure 6B, the MXene layer presents a bulk thickness of only 800 nm. The MXene nanosheets are separated when they travel from the needle tip to the roller due to the electrostatic repulsion, which forms the layered MXene structure filled with PEO/LiTFSI. The authors claimed that the atomized PEO/LiTFSI is uniformly inserted into the interlayers of the MXene nanosheets, which promotes Li^+^ diffusion. Furthermore, the abundant lithiophilic groups, such as -OH and -F, of the MXene can lower the Li nucleation energy barrier, thus the low plating potential from the CV result of the Li/MXene-PEO/SS cell.

**Table 10:** Functional MXene design at SSB interfaces.

Type of MXene	Type of SSE	Interface	Cycle Life	Interfacial Resistance	Refs.
Ti_3_C_2_T_x_	PEO	PEO/Li metal	140	NA	[152]

## 25. Summary and Future Directions

### 25.1. Cost, Scalability, and Sustainability

The future of MXenes as a viable alternative to graphite in energy storage systems hinges on their cost-effectiveness, scalability, and environmental sustainability. As MXenes move closer to commercial viability, they offer compelling opportunities for innovation in next-generation energy storage technologies. Yet, challenges such as maintaining high capacity at rapid charge/discharge rates and reducing production costs remain pivotal hurdles. These challenges, however, are also drivers of progress, prompting the development of more efficient synthesis methods and the design of MXenes with tailored electrochemical properties.

A central conclusion of this review highlights the urgent need for scalable, economically viable synthesis strategies that support the high-volume production of MXenes at competitive market prices. Establishing robust, cost-efficient manufacturing protocols is a critical step toward widespread adoption, helping to transition MXenes from laboratory-scale materials to industrially relevant components for battery systems.

Increasing the commercial availability of MXene materials would not only streamline the fabrication of energy storage devices but also enable deeper fundamental research, as researchers gain easier access to testing materials for exploration of structure–property relationships. A robust inventory of standardized MXene materials would accelerate the development of electrodes, capacitors, and multifunctional energy devices, thereby expanding the horizon of MXene-based technologies.

### 25.2. Environmental Considerations and Sustainable Synthesis

The adoption of new materials in energy storage must go hand in hand with environmental responsibility, especially as the world transitions toward low-carbon energy systems. In this context, MXenes present both opportunities and challenges. Their chemical versatility and performance advantages position them as frontrunners in the search for sustainable energy materials, but their environmental footprint during synthesis remains a concern—particularly when hydrofluoric acid (HF) is used as an etchant in traditional synthesis methods.

To address these challenges, recent research has emphasized the need to develop greener synthesis routes, including the substitution of HF with safer etchants, the reduction in water and acid usage, and the adoption of recyclable or renewable precursors. For instance, biomass-derived carbon sources, such as waste sugars or starches, can serve as environmentally benign precursors in bottom-up MXene production. In addition, metal precursors with low environmental toxicity should be prioritized to minimize ecological impact.

Particularly promising are advancements in acid recycling technologies, which aim to reduce hazardous waste and lower overall chemical consumption during MXene production. These innovations, along with efforts to minimize byproduct generation, support a more sustainable and circular approach to MXene synthesis.

### 25.3. New Material Innovations

Currently, MXenes for energy storage applications are situated at a Technology Readiness Level (TRL) of 3, indicating successful validation in controlled laboratory settings. The next critical step in their development is validation in relevant environments, which involves integrating MXenes into commercial energy storage systems under real-world operating conditions. Achieving this milestone requires cost-effective, scalable synthesis and material performance that meets or exceeds industry benchmarks for adoption.

MXenes remain in the discovery and optimization phase, with ongoing research focused on tuning functional properties—including conductivity, stability, and ion transport dynamics—to align with commercial demands. Noteworthy progress in MXene engineering includes the work of Lee et al., who demonstrated the vertical alignment of Ti_3_C_2_T_x_ MXene sheets under an in-plane electric field, a phenomenon confirmed using polarized optical microscopy and scanning electron microscopy [120]. This anisotropic alignment could be instrumental in optimizing ion pathways and enhancing device performance.

In parallel, Chen et al. introduced an innovative method to fabricate ultralow-reflection electromagnetic interference (EMI) shielding films using a magnetically aligned composite of carbonyl iron@SiO_2_ and Ni@Ag within a flexible silicone rubber matrix [154]. This breakthrough addresses challenges in flexible electronics by delivering EMI shielding predominantly through absorption rather than reflection—while maintaining mechanical resilience under tensile strain. These examples highlight the expanding multifunctionality of MXenes beyond energy storage, showcasing their adaptability across electronics and electromagnetic applications.

The structural diversity and tunability of MXenes—through variations in M elements, layer thickness, surface terminations, and interlayer spacing—introduce significant complexity into their electrochemical behavior. Each MXene exhibits unique charge storage mechanisms depending on factors such as ion size, adsorption energy, (de)intercalation barriers, ion solvation behavior, and electrolyte decomposition pathways. Additionally, the nature and distribution of surface functional groups (e.g., –OH, –F, –O) play a pivotal role in influencing capacity, stability, rate capability, and cycling performance.

Given this complexity, advancing MXene-based energy storage requires a synergistic approach that integrates theoretical modeling and experimental validation. Computational techniques such as Density Functional Theory (DFT) and machine learning/artificial intelligence (AI) can rapidly screen MXene candidates, predict ion interactions, and simulate charge storage mechanisms. When coupled with empirical validation, this hybrid framework accelerates materials discovery and optimizes time, cost, and resource investment.

This integrated strategy reflects a proactive and forward-looking effort to bridge the gap between lab-scale innovation and commercial deployment. It also highlights the transformative potential of MXenes in enabling high-performance, sustainable energy technologies. By streamlining development pipelines and reducing barriers to commercialization, MXenes are well-positioned to emerge as a pivotal material class in the future of battery innovation, flexible electronics, and multifunctional energy systems.

### 25.4. Opportunities in Next-Generation Energy Storage Technologies

MXenes have emerged as highly promising materials for next-generation energy storage systems, particularly in the advancement of solid-state batteries, due to their outstanding electrochemical performance, mechanical resilience, and surface tunability [155,156,157]. A key advantage of MXenes lies in their ability to undergo diverse surface functionalization, allowing for extensive opportunities in interfacial engineering and materials optimization.

Functionalization strategies such as polymer grafting [158], in situ growth of carbon nanotubes (CNTs), nitrogen doping [159], and the formation of hydrogen-bonded polymeric networks [160,161] have been shown to significantly enhance MXene properties. These modifications improve both electronic and ionic conductivity, capitalizing on MXenes’ intrinsically high density of charge carriers (DCC). This feature enables uniform ion transport and deposition, particularly at electrode interfaces, resulting in enhanced rate performance and cycling stability in solid-state configurations.

For example, recent studies have demonstrated that silicon–nitrogen–MXene composites, when employed as anode materials in solid-state batteries, can achieve exceptional electrochemical performance, even in the absence of a dedicated solid electrolyte. This is primarily attributed to the synergistic DCC properties of MXenes, which facilitate efficient charge transport and structural stability [162,163]—underscoring MXenes’ transformative potential in reimagining solid-state battery architectures.

### 25.5. MXenes in Solid Electrolyte and Interface Engineering

MXenes also play a crucial role as functional additives in solid-state electrolytes, particularly in composite polymer electrolytes (CPEs). Their high aspect ratio and hydrophilic surfaces disrupt polyethylene oxide (PEO) crystallinity, enhancing lithium-ion conductivity. Similar improvements have been reported in zinc-based and lithium-iodide (Li-I) solid-state systems, where MXenes contribute to the formation of multivalent ion transport channels within polymer matrices [151].

At the electrode–electrolyte interface, MXenes offer additional benefits. As electrospun interlayers between lithium metal anodes and polymer electrolytes, MXenes help lower the lithium nucleation energy barrier, leading to dendrite-free deposition and significantly improving safety—one of the central challenges in solid-state battery development. As fabrication processes become more cost-efficient, MXenes are poised to become integral to commercial solid-state battery designs, offering gains in cycle life, rate capability, energy density, and safety.

### 25.6. Materials Innovation and Sustainable Design

Further innovation can be achieved by fine-tuning MXene properties, particularly through control of surface terminations, which strongly affect performance parameters such as capacity, stability, and electrochemical kinetics [164,165,166]. Tailoring surface chemistry can enable material-specific optimizations, including compatibility with alternative battery chemistries and suppression of parasitic reactions.

MXenes also offer strategic advantages in terms of material supply resilience. Given increasing concerns over the availability and ethical sourcing of critical battery materials such as cobalt, MXenes present a path toward sustainability by allowing the substitution of more abundant and less geopolitically sensitive transition metals without significantly compromising performance. This adaptability reinforces MXenes’ role in building robust and diversified supply chains for future energy storage technologies.

### 25.7. Sustainable Synthesis Pathways

Beyond materials functionality, the sustainability of MXene production is an area of growing importance. Traditional HF-based etching methods raise concerns over toxicity and waste management. As a greener alternative, hydrothermal synthesis has gained attention for its ability to use renewable carbon sources, such as sugar-derived waste products from the food industry, to create carbon frameworks. This route not only minimizes hazardous byproducts but also significantly reduces the environmental footprint of MXene production—marking a step forward in aligning MXene technologies with green chemistry principles.

Efforts to incorporate acid recycling, solvent reuse, and low-temperature processing further contribute to the development of economically and environmentally sustainable MXene synthesis, thereby facilitating their scalable deployment.

In conclusion, MXenes represent a versatile and forward-looking platform for the development of advanced energy storage systems. Their unique combination of surface engineering flexibility, exceptional electrochemical properties, and compatibility with sustainable manufacturing techniques positions them at the forefront of innovation in solid-state batteries and beyond. Continued interdisciplinary collaboration in materials design, process engineering, and environmental science will be key to unlocking the full potential of MXenes—enabling a more efficient, resilient, and sustainable energy future.
